# Allicin in Digestive System Cancer: From Biological Effects to Clinical Treatment

**DOI:** 10.3389/fphar.2022.903259

**Published:** 2022-06-13

**Authors:** Yang Zhou, Xingxuan Li, Wenyu Luo, Junfeng Zhu, Jingwen Zhao, Mengyao Wang, Lixuan Sang, Bing Chang, Bingyuan Wang

**Affiliations:** ^1^ Department of Gastroenterology, The First Affiliated Hospital of China Medical University, Shenyang, China; ^2^ The Second Clinical College, China Medical University, Shenyang, China; ^3^ Department of Clinical Laboratory, Affiliated Hospital of Guilin Medical University, Guilin, China; ^4^ Department of Gastroenterology, Shengjing Hospital of China Medical University, Shenyang, China; ^5^ Department of Geriatric Medicine, The First Affiliated Hospital of China Medical University, Shenyang, China

**Keywords:** allicin, digestive system cancer, gastrointestinal cancer, therapy, allicin secondary metabolites

## Abstract

Allicin is the main active ingredient in freshly-crushed garlic and some other allium plants, and its anticancer effect on cancers of digestive system has been confirmed in many studies. The aim of this review is to summarize epidemiological studies and *in vitro* and *in vivo* investigations on the anticancer effects of allicin and its secondary metabolites, as well as their biological functions. In epidemiological studies of esophageal cancer, liver cancer, pancreatic cancer, and biliary tract cancer, the anticancer effect of garlic has been confirmed consistently. However, the results obtained from epidemiological studies in gastric cancer and colon cancer are inconsistent. *In vitro* studies demonstrated that allicin and its secondary metabolites play an antitumor role by inhibiting tumor cell proliferation, inducing apoptosis, controlling tumor invasion and metastasis, decreasing angiogenesis, suppressing *Helicobacter pylori*, enhancing the efficacy of chemotherapeutic drugs, and reducing the damage caused by chemotherapeutic drugs. *In vivo* studies further demonstrate that allicin and its secondary metabolites inhibit cancers of the digestive system. This review describes the mechanisms against cancers of digestive system and therapeutic potential of allicin and its secondary metabolites.

## 1 Introduction

Global cancer statistics in 2020 showed that digestive system cancers, such as colorectal cancer, liver cancer, gastric cancer, esophageal cancer, pancreatic cancer, and cholangiocarcinoma, usually have a high risk of morbidity and mortality, and digestive system cancer is considered the leading cause of cancer-related death in the world (Sung et al., 2021). Recent reports have shown that the incidence rate and mortality rate of digestive system cancer are still rising, and it has become a global health problem that seriously threatens human health (Smyth et al., 2017; Bray et al., 2018; Villanueva, 2019; Sung et al., 2021). At the same time, some studies have shown that digestive system cancers have some pathogenesis factors in common (Zhang et al., 2018; Wang et al., 2020). Therefore, comparing the pathogenesis of various digestive system cancers and the effect of anticancer agents on cancers of the digestive system may provide a basis for the discovery of more effective digestive system anti-tumor therapies. The current therapeutic strategies for digestive system cancer mainly include surgery, radiotherapy, chemotherapy and immunotherapy, which are often accompanied by many disadvantages, such as drug resistance, risk of recurrence, poor prognosis, and high cost (Pennathur et al., 2013; Gravitz, 2014; Razumilava and Gores, 2014; Dekker et al., 2019; Mizrahi et al., 2020; Smyth et al., 2020). As a result, new therapies are needed to better control digestive system cancer. To reduce the adverse effects of current major therapies, researchers have focused on natural products.

Allicin (diallyl thiosulfinate) is a natural product formed chemically on crushing a garlic clove (Sarvizadeh et al., 2021). It is unstable and transforms into a variety of bioactive secondary metabolites (Sarvizadeh et al., 2021). Allicin and its secondary metabolites are organosulfur compounds (OSCs) with a variety of biological activities, including anticancer, antioxidation, and antipathogenic activities, and their effects act on cancers of digestive system have attracted extensive attention (Min and ZhuBo, 2011; El-Saber Batiha et al., 2020). Previous studies have shown that the OSCs act against digestive system cancers by suppressing proliferation, inducing apoptosis, and inhibiting invasion, metastasis, and angiogenesis of the tumor (Sarvizadeh et al., 2021). In addition, these OSCs have been found to enhance the efficacy of chemotherapeutic drugs and reduce the side effects caused by traditional therapies under certain conditions (Zhang et al., 2020). Although the research results of many studies are promising, the findings of some experiments are controversial. For example, the results of some epidemiological investigations are inconsistent in the study of allicin intervention in gastric cancer (Kim et al., 2018; Li WQ. et al., 2019). Therefore, to further explore the anticancer effect of allicin and its possible clinical application, we searched for articles on related investigations up to March 2022 in the PubMed and Web of Science databases to systematically summarize the biological functions of these compounds *in vivo* and *in vitro* and to clarify the mechanism of the anticancer effect of allicin and its secondary metabolites on cancers of the digestive system. The aim of this review is to provide a convenient reference for researchers seeking to carry out further research.

## 2 Allicin

### 2.1 Chemical Structure and Formation of Allicin

**FIGURE 1:**
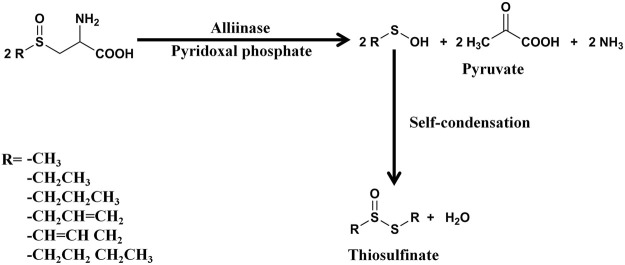
The synthesis process of thiosulfinates. One molecule of thiosulfinate is synthesized from two molecules of sulfenic acids, and the figure shows the different *R* groups of thiosulfinates (Rose et al., 2005).

**FIGURE 2:**
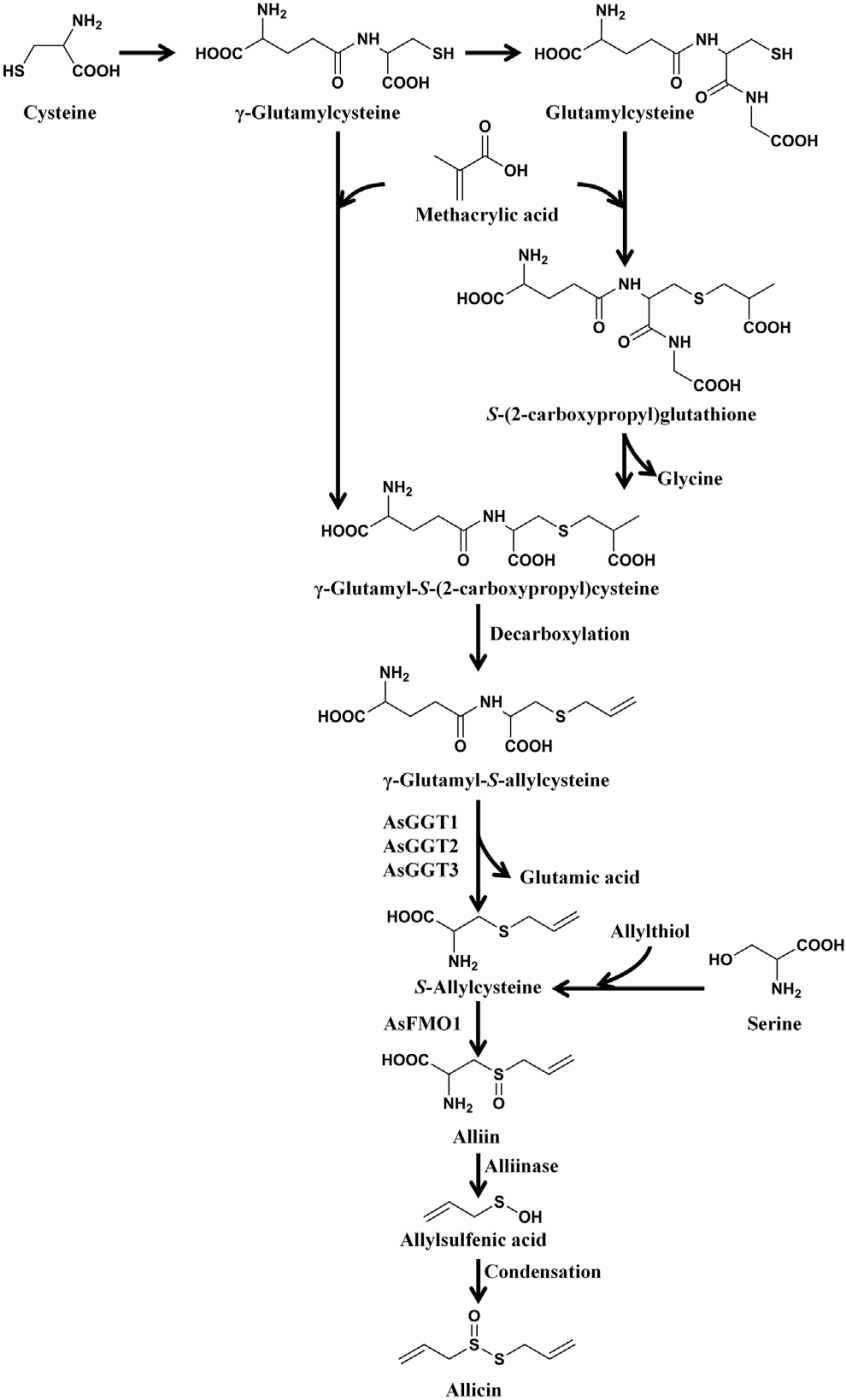
The synthesis process of alliin and allicin. Cysteine first synthesizes γ-glutamylcysteine and glutamylcysteine, and then the two compounds react with α-methacrylic acid, and synthesize γ-glutamyl-*S* (-2-carboxypropyl) cysteine and *S*-(2-carboxypropyl) glutathione, respectively. Subsequently, *S*-(2-carboxypropyl) glutathione also forms γ-glutamyl-*S* (-2-carboxypropyl) cysteine. Then γ-glutamyl-*S*-(2-carboxypropyl) cysteine undergoes decarboxylation and form γ-glutamyl-*S*-allylcysteine. γ-Glutamyl-*S*-allylcysteine first undergoes deglutamylation to produce *S*-allylcysteine (catalyzed by recombinant AsGGT1, AsGGT2, and AsGGT3), and then undergoes *S*-oxygenation (catalyzed by recombinant AsFMO1) to produce alliin. *S*-allylcysteine can also been produced by the process of serine reacting with allylthiol. After synthesis, alliin is further decomposed into allylsulfenic acid under the catalysis of alliinase, and then the two molecules of allylsulfenic acid condense spontaneously to produce one molecule of allicin. (Rose et al., 2005; Yoshimoto and Saito, 2019).

**FIGURE 3:**
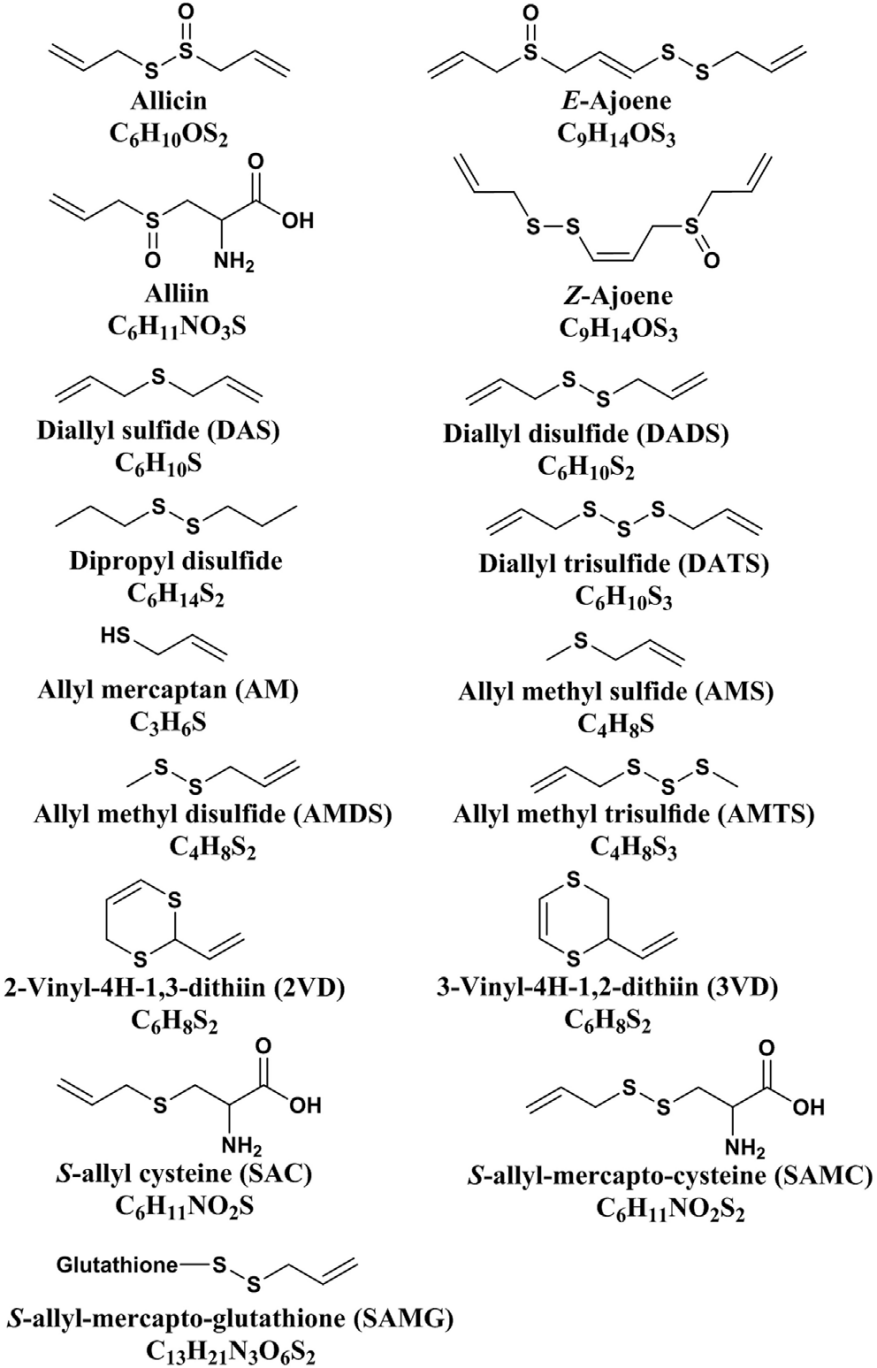
The structure of allicin and its related compounds (Zhang et al., 2020).

Allicin is the primary product formed on crushing a garlic clove. It is an electrophilic thioallyl ester of allylsulfinic acid with a pungent smell reminiscent of a pizza parlour (Borlinghaus et al., 2014). Cavallito and Bailey firstly isolated and described the properties of allicin in 1944, and Stoll and Seebeck determined its structure in 1948 (Cavallito C. J. and Bailey J. H., 1944; Stoll and Seebeck, 1948). In nature allicin is produced *via* an enzymatic reaction after plant tissue damage (Borlinghaus et al., 2014). Alliin [(+)-*S*-allyl-l-cysteine sulfoxide] is the precursor of allicin and is one of the major *S*-alk(en)yl-l-cysteine sulfoxides identified in allium plants (Stoll and Seebeck, 1948). *S*-alk(en)yl-l-cysteine sulfoxides are nonvolatile sulfur storage compounds giving rise to the different odor, flavor and biological activities in allium plants (Rose et al., 2005). They biosynthesized *via* a series of reactions as follows: *S*-alk(en)ylation of the cysteine residue of glutathione, followed by transpeptidation to remove the glycyl residue, then proceeding oxidation and loss of the glutamyl group to form the parental *S*-alk(en)yl-l-cysteine sulfoxides (Rose et al., 2005). Moreover, they can also alternatively biosynthesized *via* direct *S*-alk(en)ylation of cysteine or thioalk(en)ylation of *O*-acetyl serine followed by oxidation (Rose et al., 2005). Under the catalysis of alliinase [EC 4.4.1.4] and the presence of the cofactor pyridoxal 5′-phosphate, *S*-alk(en)yl-l-cysteine sulfoxides hydrolyze and produce pyruvate, ammonia, and sulfenic acids (Block, 1992). Sulfenic acid (RSOH) synthesized from *S*-alk(en)yl-l-cysteine sulfoxides are highly reactive and thus converted into thiosulfinates by self-condensation, and due to different R groups, a variety of thiosulfinates can be produced (Figure 1) (Yoshimoto and Saito, 2019). Thiosulifnates derived from allium plants can be divided into four types: 1) fully saturated, RS(O)SR' (R,R' = Me or Pr); 2) mono- or bis-β,γ-unsaturated thiosulfinates AllS(O)SMe, AllSS(O) Me or AllS(O)SAll; 3) mono-α,β-unsaturated thiosulfinates; and 4) mixed α,β- and β,γ-unsaturated thiosulfinates (Block, 1992). As a major *S*-alk(en)yl-l-cysteine sulfoxides, the main processes of alliin synthesis are the same as the process described above in general. γ-Glutamylcysteine and glutamylcysteine produced by cysteine are the starting compounds for the synthesis of alliin. With the participation of α-methacrylic acid, both γ-glutamylcysteine and glutamylcysteine can form γ-glutamyl-*S*-(2-carboxypropyl) cysteine (Rose et al., 2005). γ-Glutamyl-*S*-(2-carboxypropyl) cysteine undergoes decarboxylation and form γ-glutamyl-*S*-allylcysteine (Rose et al., 2005). In garlic, γ-glutamyl-*S*-allylcysteine first undergoes deglutamylation to produce *S*-allylcysteine, which is catalyzed by recombinant proteins AsGGT1, AsGGT2, and AsGGT3 (Yoshimoto and Saito, 2019). Moreover, *S*-allylcysteine can also been produced *via* the process of serine reacting with allylthiol. Then *S*-allylcysteine undergoes *S*-oxygenation which is catalyzed by recombinant AsFMO1 protein, to produce alliin (Yoshimoto and Saito, 2019). Alliin is further decomposed into allylsulfonic acid under the catalysis of alliinase, and then the two molecules of allylsulfenic acid can condense spontaneously to produce one molecule of allicin, which is a di-*S*-β,γ-unsaturated thiosulfinate [AllS(O)SAll] (Block, 1992). The specific synthesis process of alliin and allicin are shown in Figure 2. Allicin can be further decomposed into allylsulfenic acid and thioacrolein (Rose et al., 2005). Thioacrolein can undergo self-condensation by a Diels–Alder reaction to produce the cyclics 2-vinyl-[4H]-1,3-dithin (2VD) and 3-vinyl-[4H]-1,2-dithin (3VD), and allylsulfenic acid can also undergo self-condensation to form allicin again (Block, 1985). Allicin is a liposoluble OSC that is unstable after synthesis, and *in vitro*, it will immediately decompose into a series of liposoluble organic sulfides, including diallyl disulfide (DADS), diallyl trisulfide (DATS), ajoene, allyl methyl trisulfide (AMTS), dithiins, and diallyl sulfide (DAS) (El-Saber Batiha et al., 2020). *In vivo*, allicin can also synthesize water-soluble OSCs such as *S*-allylmercaptocysteine (SAMC) and *S*-allylmercaptoglutathione (SAMG) by interacting with l-cysteine and glutathione (GSH), respectively (Rouf et al., 2020). The structure of allicin and its related compounds are shown in Figure 3.

In addition, allicin can undergo the thiol-disulfide exchange reaction (TDER) with protein thiols, which is considered to be the key to allicin’s biological activity (Poole, 2015). The TDER is that thiols (RSH) react with disulfides (R'SSR') and form a new disulfide (RSSR′) and a new thiol (R'SH) (Rose et al., 2018). In the TDER, though both the divalent sulfur atom and the sulfinyl sulfur atom of allicin are electrophilic, most of the chemistry occurs by nucleophilic attack on the divalent sulfur, with the sulfinyl sulfur being the leaving group when the *O*-atom is protonated (Borlinghaus et al., 2021). The polarized bond between the *O*- and *S*-atoms of the sulfinyl group significantly weakens the disulfide bond in allicin, making it more reactive to target nucleophilic thiol groups than a simple disulfide bond, and the newly formed disulfide bond can be reduced back to a thiol just like other protein disulfide (Borlinghaus et al., 2021). Allicin acts as a sulfenylating agent of thiols to form disulfides with the formation of water. One molecule of allicin can totally react with two molecules of thiols and form two molecules of disulfides (Poole, 2015). This is because the leaving group in the first exchange between thiol and allicin generates allylsulfenic acid, which can also act as an electrophilic sulfenylating agent towards a thiol with the expulsion of water (Poole, 2015). In addition, the allylsulfenic acid can dimerize to generate allicin and water again (Block, 1992). Therefore, allicin is a crucial thiol oxidant participating in the thiol/disulfide homeostasis. Based on the chemistry, allicin can react with protein thiols, modify Cys residues, thereby regulating various metabolic processes in cells and exert its biological activity (Poole, 2015).

In addition to natural plants, allicin and its secondary metabolites also exist in some artificial preparations, such as aged garlic extract (AGE), aged black garlic extract (ABGE), garlic powder (GP), and garlic oil (GO). The commercially available “Allimaxˮ is stabilized allicin, and is extracted from fresh, raw garlic through crushing, filtration and temperature controlled extraction process *via* a patented aqueous extraction method, and the pure allicin is dissolved in water (Josling, 2001). GP contains alliin and a small amount of oil-soluble sulfur compounds, and its physiological activity is similar to that of fresh garlic (Lawson and Gardner, 2005). The biologically active compounds in GO are oil-soluble sulfur compounds such as DADS and DATS (Amagase et al., 2001). AGE consists of various OSCs, both hydrophilic and hydrophobic, and mainly contains water-soluble compounds (e.g., SAC, SAMC, and GSAC) with a small amount of oil-soluble sulfur compounds (e.g., DASn) (Amagase et al., 2001; Kodera et al., 2020). ABGE is a kind of AGE that is made of aged black garlic, and it plays a role in anticancer activity, regulates lipid metabolism, anti-inflammation, and antioxidant activities and is more potent than other preparations in many of the therapeutic properties of garlic (Dong et al., 2014; Nam et al., 2018; You et al., 2019). As a result, many researchers use these kinds of garlic preparations as materials to study the biological functions of allicin and its secondary metabolites.

### 2.2 Toxicity

Allicin is a membrane-permeable compound that can enter cells easily and can oxidize cellular thiols such as glutathione or cysteine residues in proteins as well as enzymes containing reactive cysteine since it is a reactive sulfur species (RSS) (Miron et al., 2000; Gruhlke and Slusarenko, 2012). Oxidation of protein thiols results in changes in protein structure, for example through disulfide bond formation (Gruhlke and Slusarenko, 2012), which in turn leads to changes in cell function, which may explain its cytotoxic potential and toxicity on normal cells (Gruhlke et al., 2016).

A randomized controlled trial has shown that high doses of allicin in sensitive people can cause a range of side effects, including insomnia, vomiting, heartburn, dizziness, diarrhea, tachycardia, nausea, bloating, flushing, headache, mild orthostatic hypotension, sweating, offensive body odor, and flatulence (Desai et al., 1990). Another experiment showed that low doses (250, 500 mg/kg/day) (*p* < 0.02) of garlic endogenous enhanced antioxidant status in mice, but high doses (1,000 mg/kg/day) induced pathological changes in morphology of kidney and liver, indicating dose-related toxicity (Banerjee et al., 2001). Other researches have shown that low doses of garlic are safe, whereas therapeutic doses might cause mild gastrointestinal disorders, while high doses have been reported to cause liver damage (Almogren et al., 2013; Ried and Fakler, 2014). Besides, an *in vivo* study showed that high doses of raw garlic over a long period of time can lead to weight loss, as well as red blood cell lysis, which could be associated with oxidative hemolysis (Borrelli et al., 2007). Chronic administration of garlic powder (50 mg/d) also resulted in inhibition of spermatogenesis in rats (Dixit and Joshi, 1982). Moreover, intraperitoneal and oral administration of high doses (5 ml garlic juice/kg) led to weight loss, and hepatic and pulmonary toxicity in rats (Nakagawa et al., 1980). Depending on the dose consumed, symptoms of garlic poisoning usually appear a day or several days after taking (Lee et al., 2006).

### 2.3 Biological Function of Allicin and its Secondary Metabolites

#### 2.3.1 Anticancer

An early study in 1960 showed that tumor growth could be inhibited by intraperitoneal injection of tumor cells into mice after culturing ascites with allicin *in vitro* (Dipaolo and Carruthers, 1960). Allicin can induce cancer cells to form apoptotic bodies and DNA ladders (Oommen et al., 2004). Furthermore, it has been found that allicin can induce redox shifts in cultured human cells, which results in the activation of the mitochondrial apoptotic pathway (Miron et al., 2008). In addition, studies found that NF-E2-related factor-2 (Nrf2) can mediate the apoptosis induced by allicin in colonic cancer cells, although Nrf2 is usually considered an anti-apoptotic factor that upregulates the anti-apoptotic protein Bcl-2 (Bat-Chen et al., 2010; Niture and Jaiswal, 2012). As the precursor of allicin, alliin shows anti-proliferative potential in a human gastric adenocarcinoma cell line, and it has been confirmed that it does not affect the growth of normal intestinal cells when inducing apoptosis of gastric cancer cells (Mansingh et al., 2018). In addition, DADS has been proven to inhibit human esophageal xenograft tumors through various pathways, such as RAF/MEK/ERK and mitochondria-dependent pathways (Yin et al., 2014). DATS works against tumors by blocking the cell cycle, inhibiting tumor cell proliferation, and inhibiting angiogenesis (Jiang X. et al., 2017; Wei et al., 2017). *Z*-ajoene can inhibit the growth of tumor cells by resisting proliferation, stimulating apoptosis, and increasing the production of peroxide, caspase-3-like, and caspase-8 (Li et al., 2002; Bayan et al., 2014). SAMC and SAC are also two essential anticancer allicin secondary metabolites that play antitumor roles by inhibiting tumor cell proliferation and inducing apoptosis (Xu et al., 2014; Zhang et al., 2014; Xiao et al., 2018).

Allicin not only protects against tumors but also alle*via*tes the adverse effects of anticancer treatment and enhances the chemotherapeutic response under certain conditions. For instance, allicin has a protective effect against liver toxicity induced by As_2_O_3_ (arsenic trioxide), an effective therapeutic agent for acute promyelocytic leukemia, through the activation of the Nrf2 signaling pathway involving KLF9 in rats (Yang et al., 2017). In addition, a study suggests the beneficial role of allicin as an adjuvant to TAM (tamoxifen, which is widely used for the treatment of hormone-dependent breast cancer) in cancer treatment by alleviating liver injury (Suddek, 2014).

We summarize the effects of allicin and its secondary metabolites against digestive system cancer in Table 1. In addition to allicin, DATS has also shown noticeable anti-cancer effects in digestive system. Therefore, in sections 4.2 and 4.3 of this review, we compare the anti-cancer effects, stability, action targets and specific mechanisms of the two compounds in detail.

**TABLE 1:** Summary of mechanisms of allicin and its secondary metabolites against cancers of digestive system.

Cancer	Intervention reagent	Dose(s)	Effects	Type of research	Animal model	Cell lines	References
Gastric cancer	Allicin	NA	Increase Bax and Fas expression, and decrease Bcl-2 expression level	Clinical trial	NA	NA	Zhang et al. (2008)
	Allicin	3, 6, 9, and 12 μg/ml	Induce gastric cancer cell stagnation at M stage and up-regulated *p21WAF1* and *p16INK4* genes	*In vitro*	NA	MGC-803 and SGC-7901	Ha and Yuan, (2004)
	Allicin	3, 6, and 12 mg/L	Arrest the G2/M phase, inhibited cell proliferation and induced apoptosis	*In vitro*	NA	SGC-7901	Tao et al. (2014)
	Allicin	15–120 μg/ml	Simultaneously active intrinsic mitochondrial and extrinsic Fas/FasL-mediated pathways of apoptosis, induce cytochrome C release from the mitochondria, increase caspase-3, -8, and -9 activation, upregulate Bax and Fas expression in the tumor cells	*In vitro*	NA	SGC-7901	Zhang et al. (2010)
	Allicin	0.1, 0.05, and 0.016 mg/ml	Inhibit telomerase activity and induce apoptosis	*In vitro*	NA	SGC-7901	Sun and Wang, (2003)
	Allicin	0.1, 1, and 10 μg/ml	Induce apoptosis through the P38-MAPK/caspase-3 signaling pathway	*In vitro*	NA	MGC-803, BGC-823 and SGC-7901 cell	Zhang et al. (2015)
	ABGE	*In vivo*: 0, 200, 400, 800 mg/kg, intraperitoneally, for 2 weeks; *in vitro*: 0, 10, 50, and 100 mg/ml	Induce apoptsis of cancer cells and inhibit the growth of tumor	*In vivo* and *in vitro*	Tumor-bearing mice model	SGC-7901	Wang et al. (2012)
	DATS	*In vivo*: 20, 30 and 40 mg/kg; *in vitro*: 0, 25, 50, 100, 200, 400 µM	Decrease G1 phase, increase G2/M phase, induce apoptosis by down-regulating Bcl-2 and activating MAPK and affecting PI3K/AKT pathways, increase levels of IL-12, IFN-γ and TNF-α level in the host, suppress tumor invasion and metastasis	*In vivo* and *in vitro*	SGC-7901 xenograft mice model	SGC-7901	Jiang et al. (2017b)
	DATS	*In vivo*: 20, 30 and 40 mg/kg; *in vitro*: 50, 100, and 200 μmol/L	Induce G2/M phase cell cycle arrest, down-regulate Bcl-2 as well as up-regulate Bax, P53 and cytochrome C, induce apoptosis through activation of the caspase pathway, attenuate Nrf2/Akt and activative of the JNK and P38-MAPK pathways, and improve the anti-tumor efficacy of cisplatin (DDP)	*In vivo* and *in vitro*	BALB/c nude mice BGC-823 xenograft model	BGC-823	Jiang et al. (2017a)
Colorectal cancer	Allicin	In the mouse model: 48 mg/kg to achieve 5 g/day; in HCT-116 cells: 25 µM for 24 h	Prevent tumorigenesis by inhibiting the STAT3 signaling pathway activation	*In vivo* and *in vitro*	AOM/DSS model of colorectal cancer mouse model	HCT-116	Li et al. (2019b)
	Allicin	0, 2, 4, 8, 16, 32, 64, 128 and 256 μg/ml	Improve the radiosensitivity of colorectal cancer cells by inhibiting NF-κB signaling pathway	*In vivo* and *in vitro*	Transplantation of CT26 cell in BALB/c mice	HCT-116,CT26	Huang et al. (2020)
	Allicin	10–25 µM	Transiently deplete the intracellular GSH level, and inhibit the proliferation of cancer cells	*In vitro*	NA	HT-29	Hirsch et al. (2000)
	Allicin	0–1.2 mM	Reduce cell viability and cell proliferation	*In vitro*	NA	HT-29	Gruhlke et al. (2016)
	Allicin	1–50 μg/ml for 24, 48, and 72 h	Induce apoptotic death *via* Nrf2, enhance hypodiploid DNA content, decrease Bcl-2, increase Bax and capability of releasing cytochrome C from mitochondria to cytosol	*In vitro*	NA	HCT-116, LS174T, HT-29, and Caco-2	Bat-Chen et al. (2010)
	Allicin	3 and 6 μg/ml	Inhibit invasion and metastasis at non-cytotoxic concentration *via* down-regulating the expression of VEGF, u-PAR and HPA mRNA	*In vitro*	NA	LoVo	Gao et al. (2009b)
	Allicin	4 and 8 mg/L	Inhibit cancer cells proliferation by induction of apoptosis and arrestment of cell cycle, and enhancing the cytotoxicity of CPT-11	*In vitro*	NA	LoVo	Gao et al. (2009a)
	Allicin	1.625, 3.125, 6.25, 12.5, 25, 50, and100 µM	5-FU combined with allicin has a synergistic effect against colon cancer cells, and better results can be obtained than the single-agent treatment at IC50 with a lower concentration of 5-FU.	*In vitro*	NA	DLD-1	Țigu et al. (2020)
	Allicin	2.5, 5, 10, 25, 50, 75, and 100 μg/ml	Enhanced the effects of 5-FU and oxaliplatin against cancer cells	*In vitro*	NA	Caco-2 and HT-29	Perez-Ortiz et al. (2020)
	ABGE	0, 20, 50, and 100 mg/ml	Inhibit the growth and induced apoptosis in HT29 cells *via* inhibiting of the PI3K/Akt pathway	*In vitro*	NA	HT-29	Dong et al. (2014)
	AGE	Active treatment: high-dose AGE 2.4 ml/d; controlled group: low-dose AGE 0.16 ml/d	AGE can reduce the occurrence and growth and spread of colorectal adenomas	Clinical trial	NA	NA	Tanaka et al. (2006)
	AGE	0, 0.1, 1, and 10 mg/ml	Inhibit proliferation and angiogenesis through the suppression of endothelial cell motility, proliferation, and tube formation	*In vitro*	NA	HT-29, SW480, and SW620	Matsuura et al. (2006)
	AGE	*In vivo*: a basal diet containing 3% wt/wt AGE; *in vitro*: 0, 1, 5, or 10 mg/ml AGE	Suppress the proliferative activity in adenoma and adenocarcinoma lesions, without effect on normal colon mucosa, delay cell cycle progression by downregulating cyclin B1 and cdk1 expression *via* inactivation of NF-κB but did not induce apoptosis	*In vivo* and *in vitro*	F344 rats with DMH-induced colon carcinogenesis	DLD-1	Jikihara et al. (2015)
	CGE	0.125, 0.25, 0.5, or 1 μg/ml	Inhibit proliferation, induces arrest of cell cycle and apoptosis	*In vitro*	NA	Caco-2	Bagul et al. (2015)
	DADS	62.5, 125, 250, 500, and 1,000 ppm	Increase activities of phase II enzymes such as GST, NAD(P)H-dependent quinone reductase, and UDP-glucuronosyl transferase in the liver and colon	*In vivo*	AOM-induced colon caicinogenesis in male F344 rats	NA	Reddy et al. (1993)
	DADS	1 mg thrice weekly; 0.5 mg thrice weekly	Reduce the toxicity of 5-FU and inhibit the growth of human colon tumor cell xenografts	*In vivo*	NCr nu/nu mice xenotransplanted colon cancer cell line HCT-15	HCT-15	Sundaram and Milner, (1996)
	DADS	85 ppm of DADS (60 mg daily human equivalent dose) in the diet	Inactivate NF-κB and prevent colitis-induced colorectal cancer by inhibiting GSK-3β	*In vivo*	FVB/N mice treated with AOM/DSS	NA	Saud et al. (2016)
	DAS	200 mg/kg	Reduce the incidence rate of colorectal adenocarcinoma	*In vivo*	C57BL/6J mice with DMH-induced colorectal cancer	NA	Wargovich, (1987)
	DATS	1–100 µM	Suppress the proliferation and induces apoptosis through oxidative modification of β-tubulin	*In vivo* and *in vitro*	Nude mice model bearing HCT-15 xenografts	HCT-15 and DLD-1	Hosono et al. (2005)
	SAMC	0–450 µM	Inhibit cell proliferation and induce apoptosis *via* the JNK and P38 pathways	*In vitro*	NA	SW620	Zhang et al. (2014)
	*Z*-ajoene	0, 10, and 30 µM	Inhibit growth of colon cancer cells by promotion of CK1α dependent β-catenin phosphorylation	*In vitro*	NA	SW480	Li et al. (2020b)
Liver cancer	Allicin	*In vivo*: 5 mg/kg/day, every 2 days for 3 weeks; SK-Hep-1 cells: 0, 1, 2, 4, 8,10, 16, 20, 32, 40, and 64 μg/ml; BEL -7402: 0, 1.25, 2.5, 5, 10, 20, 40, 80,and 160 μg/ml	Promote anti-tumor activity of 5-FU through ROS-mediated mitochondrial pathway	*In vivo* and *in vitro*	HCC xenograft tumors in nude mice	SK-Hep-1 and BEL-7402	Zou et al. (2016)
	Allicin	0, 15, 20, 25, 35, 40, and 50 µM	Induce apoptosis through caspase-dependent and caspase-independent pathways by ROS overproduction	*In vitro*	NA	Hep G2 and Hep 3B	Chu et al. (2012)
	Allicin	35 µM	Induce P53-mediated autophagy, decrease cytoplasmic P53, the PI3K/mTOR signaling, and the level of Bcl-2, increase the expression of AMPK/TSC2 and Beclin-1	*In vitro*	NA	Hep G2	Chu et al. (2013)
	Allicin	5–100 μM	Reduce the aflatoxin B1 genotoxicity in Hep G2 cells	*In vitro*	NA	Hep G2	Belloir et al. (2006)
	SAC	*In vivo*: 1 mg/kg/day; *in vitro*: 0–40 mM	Suppress proliferation and metastasis of hepatocellular carcinoma	*In vivo* and *in vitro*	Orthotopic xenograft liver tumor model	MHCC97L	Ng et al. (2012)
	SAC	5–100 μM	Reduce the aflatoxin B1 genotoxicity and the DNA damage induced by DMN in Hep G2 cells	*In vitro*	NA	Hep G2	Belloir et al. (2006)
	AGE	500 mg/day	Prevent a decline of NK cell number and activity in patients with advanced cancer	Clinical trial	NA	NA	Ishikawa et al. (2006)
	AGE	5% w/v, 0.5 ml daily	Against hepatotoxicity, oxidative stress and the hepatocarcinoma induced by p-dimethylaminoazobenzene and phenobarbital in the experimental rats	*In vivo*	*Rattus norvegicus* fed chronically with two liver carcinogens, p-dimethylaminoazobenzene and phenobarbital to produce hepatotoxicity	NA	Pathak et al. (2020)
	Alliin	NA	Reduce DNA damage induced by NDMA in liver	*In vitro*	DNA damage induced by NDMA in SPF rat liver	NA	Singh et al. (2006)
	AM	5–100 μM	Decrease the DNA damage induced by DMN in Hep G2 cells	*In vitro*	NA	Hep G2	Belloir et al. (2006)
	DADS	5–100 μM	Reduce the aflatoxin B1 genotoxicity and benzo(a)pyrene genotoxicity in Hep G2 cells	*In vitro*	NA	Hep G2	Belloir et al. (2006)
	DADS	100 μmol/L	Induced apoptosis through P38-MAPK and caspase-3	*In vitro*	NA	Hep G2	Ji et al. (2010)
	DAS	5–100 μM	Reduce the aflatoxin B1 genotoxicity, and show a low effect towards DMN genotoxicity in Hep G2 cells	*In vitro*	NA	Hep G2	Belloir et al. (2006)
	SAMC	300 mg/kg	Inhibit hepatocarcinogenesis through targeting LRP6/Wnt pathway	*In vivo*	Xenograft and orthotopic HCC nude mice model	HuH-7	Xiao et al. (2018)
Cholangiocarcinoma	Allicin	0, 5, 10, 20, and 40 µM	Inhibit cell proliferation and invasion through STAT3 signaling	*In vivo* and *in vitro*	Nude mouse model of CCA	HuCCT-1 and QBC939	Chen et al. (2018)
Esophageal cancer	DAS	200 mg/kg	Inhibit the tumorigenic effects of potent, metabolically activated monoalkylating carcinogens in the gastrointestinal tract	*In vivo*	DNA-damaging and tumorigenic effects induced by NMBA in rat esophagus	NA	Wargovich et al. (1988)
	Ajoene	NA	Inhibit proliferation and induce apoptosis of human esophageal-cancer cells	*In vitro*	NA	WHCO1	Kaschula et al. (2016)
	Ajoene analogue	10 µM	Suppress cell proliferation, induce G2/M cell cycle arrest, and induce apoptosis *via* caspase-3 activation	*In vitro*	NA	WHCO1	Kaschula et al. (2012)
	Ajoene analogue	NA	Induce cytotoxicity by activating the unfolded protein response *via* CHOP/GADD153	*In vitro*	NA	WHCO1	Siyo et al. (2017)
Pancreatic cancer	Allicin	10 mg/kg	Inhibit tumor growth and prolonged survival time	*In vivo*	C57/BL6 nude mice pancreatic cancer xenograft model	BXPC-3	Wang et al. (2013)
	DATS	100 μmol/L	Induces apoptosis of pancreatic tumorigenic cells and ductal epithelial cells	*In vitro*	NA	Capan-2, and H6C7	Ma et al. (2014)
	Garlic oil	2.5 and 10 µM	Induce pro-apoptosis effects on AsPC-1 cells in a dose- and time-dependent manner	*In vitro*	NA	AsPC-1, PANC-1, and Mia PaCa-2	Lan et al. (2013)

#### 2.3.2 Against Pathogenic Organisms

Garlic has long been used as an antimicrobial agent, and allicin is considered to be the primary substance that contributes to garlic’s antimicrobial activity (Cavallito C. J. and Bailey J. H., 1944). In addition to having a wide range of antibacterial and antifungal properties, allicin has also been found to have antiviral and antiparasitic effects (Guo, 2014).

##### 2.3.2.1 Antibacterial Effect

Allicin can inhibit the growth of both Gram-negative and Gram-positive bacteria, G(-) bacteria such as *Bacillus* spp. and *Streptococcus* spp. and G(+) bacteria such as *Salmonella typhimurium*, *Agrobacterium tumefaciens*, *Escherichia coli* K12, *Vibrio cholera*, *Pseudomonas syringae* (various pathovars) (Cavallito C. J. and Bailey J. H., 1944; Small et al., 1947; Feldberg et al., 1988; Curtis et al., 2004; Leng et al., 2011). In preclinical trials, allicin has been proven to protect against *Helicobacter pylori* (HP), which is considered to be an important factor for gastritis, peptic ulcers and gastric cancer, but clinical studies have shown that taking fresh oral garlic and using garlic oil cannot improve HP infection in most conditions (Graham et al., 1999; Aydin et al., 2000; Leontiev et al., 2018). A meta-analysis showed that allicin can enhance the effect of anti-HP when combined with front-line treatment, PPI triple therapy or bismuth-containing quadruple therapy (Si XB. et al., 2019). Allicin can also protect against some drug-resistant strains, such as *Staphylococcus aureus* (NBRC 12732) and *Staphylococcus aureus* (clinical isolates) (Cutler and Wilson, 2004; Fujisawa et al., 2009). In addition, allicin has also been found to enhance the antimicrobial effect when treated combined with other antibiotics (Choo et al., 2020).

The antibacterial activity is based on allicin’s two essential features: entering the bacterial cell and killing it (Borlinghaus et al., 2014). Due to its lipophilic character, allicin can easily diffuse across both natural and artificial phospholipid membranes, which means that allicin can easily enter the cell (Miron et al., 2000). It is noteworthy that similar to penicillin, citrinin, gliotoxin, clavacin and pyocyanines, the antibacterial activity of allicin can be suppressed by interacting with cysteine (Cavallito CJ. and Bailey JH., 1944). It has been reported that allicin inhibits the proliferation of bacteria by its–S(O)–S–group because it can react with the sulfhydryl group of cellular proteins to form mixed disulfides (Kyung, 2012). In addition, allicin can inhibit the synthesis of DNA, RNA, and protein in bacteria, and the inhibition effect on RNA synthesis is more significantly (Feldberg et al., 1988). Diallyl polysulfides are the decomposition products of allicin. Studies have shown that diallyl polysulfides can modify bacterial cell membrane or cell wall by reacting with sulfhydryl groups to disrupt the composition and integrity of bacterial cell membrane or cell wall (Lu et al., 2011).

##### 2.3.2.2 Antifungal Effect

It has been reviewed that allicin can inhibit the growth of various fungi ranging from yeasts to filamentous fungi, while in another review, allicin has been suggested to be the main antifungal compound in garlic; moreover, the secondary metabolites of allicin can stimulate cellular immunity and have better effects than conventional chemotherapy in the antifungal process (Davis, 2005; Choo et al., 2020). In addition, studies have shown that allicin can protect against *Candida albicans*, and its potency is comparable to that of fluconazole both *in vitro* and in a systemic candidiasis mouse model (Khodavandi et al., 2011). Recently, allicin has been reported to show more effective inhibition of the growth of yeast BY4742 cells than of bacteria (Leontiev et al., 2018). *YKL071w* gene in *S. cerevisiae* is highly induced by allicin and other thiol-reactive compounds, and in silico analysis revealed multiple Yap1p binding motifs in the YKL071w promoter sequence (Yu et al., 2010). Allicin has been found to directly activate Yap1p, which is considered to be the central regulator of the S. cerevisiae oxidative stress response, target at the C-term C598 and C620 residues to play an antifungal role (Gruhlke et al., 2017). The antifungal effect of allicin is closely related to its chemical structure. The reactivity of thiosulfinates towards thiol-groups is important for their antimicrobial activity (Small et al., 1947; Wills, 1956). The chemical basis of the reaction is that electron-withdrawing effect of the *O*-atom creates an electrophilic sulfur centre which reacts readily with thiols, thereby forming an *S*-thioallyl adduct (Leontiev et al., 2018). Therefore, allicin affects the fungal enzymes and proteins with thiol-groups through its proteotoxicity. Moreover, it has also been found that combining allicin with antifungal agents can enhance the antifungal effect; for example, allicin synergizes with amphotericin B against *Candida albicans* by enhancing its oxidative damage effect (Shen et al., 1996; An et al., 2009).

##### 2.3.2.3 Antiviral Effect

The antiviral activity of allicin and its secondary metabolites, such as ajoene, DATS, DAS, and DADS, has been proven both *in vivo* and *in vitro* (Weber et al., 1992; Walder et al., 1997; Fang et al., 1999; Liu et al., 2004; Terrasson et al., 2007; Hall et al., 2017; Wang et al., 2017). The crude extract of garlic and thiosulfinates have been found to be more active on the envelope virus (herpes simplex virus-1 and 2, parainfluenza-3, vaccinia virus, vesicular stomatitis virus) than non-enveloped virus (human rhinovirus-2) (Weber et al., 1992). The molecular mechanism is that OSCs react with the thiol group in various active viral proteins or enzymes that are crucial for microbial surveillance and fusion (Ankri and Mirelman, 1999; Jain et al., 2007). Allicin has been found to inhibit the viral RNA polymerase by react with the thiol groups, and has been proven to protect against REV in specific pathogen-free chickens by reducing the immunosuppression induced by REV through the ERK/mitogen-activated protein kinase pathway (Schafer and Buettner, 2001; Wang et al., 2017). DATS, the secondary metabolite of allicin, has been found to fight against CMV both *in vivo* and *in vitro* by inhibiting viral replication and reducing the DNA load of HCMV in mice (Liu et al., 2004; Zhen et al., 2006).

##### 2.3.2.4 Antiparasitic Effect

Allicin also plays a role in inhibiting the growth of various kinds of parasites, such as *Schistosome*, *Babesia*, *Theileria equi*, *Plasmodium falciparum*, and *Trypanosoma brucei* (Waag et al., 2010; Salama et al., 2014; Metwally et al., 2018). In a study conducted by Coppi *et al.*, allicin was shown to inhibit malaria infection by inhibiting both sporozoite infectivity and erythrocytic stages (Coppi et al., 2006). Their studies showed that allicin can inhibit the cleavage of CSP, which is the main surface protein of Plasmodium sporozoites and has cell invasion activity (Coppi et al., 2005; Coppi et al., 2006). In addition, the studies conducted by Salama et al. show that allicin has a potent effect against *Babesia* parasites, and it is speculated that the inhibition may occur at the invasion step (Salama et al., 2014). Moreover, they found that combining allicin with diminazene aceturate improves antiparasitic effect (Salama et al., 2014). Recently, a study found that mice treated with allicin can increase the expression of IL-13, which is a cytokine that has an antiparasitic effect, and this study indicated that allicin protects against *Schistosomiasis* through its anti-inflammatory and immunoregulatory effects (Metwally et al., 2018).

#### 2.3.3 Affect Gut Microbiota

In addition to against bacteria, allicin has also been shown to modulate the composition of gut microbiota (GM) and increase the diversity of beneficial bacteria in animal models (Guillamón et al., 2021). Allicin has been found to play a role in improving intestinal epithelial barrier function and preventing barrier damage *via* a microbiota-regulated short-chain fatty acid-TLR4/MyD88/NF-κB cascade response in an acrylamide-induced rat model (Yuan et al., 2021; Gao et al., 2022). Researchers suggested that allicin could block intestinal bacterial translocation by increasing the immunologic barrier function of mesenteric lymph nodes by modulating dendritic cell maturation (Zhang Y. et al., 2017). Moreover, in alcoholic hepatic steatosis mice, allicin has been found to modulate the GM and improve the CD14-TLR4 pathway to alleviate inflammation in the liver (Panyod et al., 2020).

#### 2.3.4 Antioxidation

As early as 2006, through detailed kinetic and mechanistic studies, allicin was confirmed to have strong antioxidant properties (Okada et al., 2006). Allicin is a reactive sulfur species (RSS) and a potent thiol-trapping reagent, rapidly reacting with glutathione (GSH) to yield *S*-allylmercaptoglutathione (GSSA) (Borlinghaus et al., 2021). Thus, allicin depletes the intracellular GSH pool and reacts with cysteine thiols available in proteins through *S*-thioallylation (Borlinghaus et al., 2021). This reaction is the key to the biological activity of allicin, and the reversible oxidation and reduction of protein-thiols is the core of many processes in cells (Schafer and Buettner, 2001). However, some studies have found opposite results (Horev-Azaria et al., 2009; Izigov et al., 2011). Allicin has been observed to up-regulate the intracellular glutathione level, which may be related to the antioxidant and SH-modifying properties of its derivatives, *S*-allylmercaptocysteine (CSSA), and *S*-allylmercaptoglutathione (GSSA) (Horev-Azaria et al., 2009; Izigov et al., 2011). More research is needed to explain the paradoxical effects. Allicin is often described as an antioxidant, with the reasons from following two aspects (Borlinghaus et al., 2021). First, chemically, allicin readily undergoes a Cope elimination reaction at room temperature to form allylsulfenic acid (Vaidya et al., 2009). Allylsulfenic acid is a very potent antioxidant, and two molecules of allylsulfenic acid can be converted into allicin again by self-condensation (Block, 1992). Secondly, allicin induces mild oxidative stress in cells which activates oxidative stress protection responses and makes cells more resistant to subsequent greater oxidative damage (Borlinghaus et al., 2021). In a hypertrophic heart mouse model, the clearance of intracellular ROS by allicin was measured, and has been shown to reduce the production of ROS and block ROS-dependent ERK1/2, JNK1/2, AKT, NF-κB and Smad signaling, which leads to the inhibition of hypertrophy (Liu et al., 2010). Another study showed that allicin can stimulate the inhibition of acrylamide-induced oxidation by regulating the mitogen-activated protein kinase (MAPK) pathway in BRL-3A cells (Hong et al., 2019). The antioxidant properties of allicin have been used to protect human umbilical vein endothelial cells (HUVECs) from oxidative stress and senescence induced by hydrogen peroxide and to improve the quality of aged oocytes *in vitro* (Zhang M. et al., 2017; Park et al., 2019). Allicin was also used to protect neurons from glutamate-induced oxidative stress, which suggested that allicin may be an effective treatment strategy for spinal cord injury (Liu et al., 2015). In addition, allicin can protect nucleus pulpotheca cells from oxidative stress and mitochondrial dysfunction induced by advanced oxidative protein products by inhibiting the P38-MAPK pathway (Xiang et al., 2020). Moreover, allicin has been shown to attenuate depressive like behaviors triggered by long-term high-fat diet consumption, which is associated with sustained oxidative stress damage and insulin resistance (Gao et al., 2019b). It has been found to improve the behaviors by inhibiting ROS production and oxidative stress, improving mitochondrial function, regulating autophagy, and reducing insulin resistance in the hippocampus *via* optimization of the NOX/Nrf2 imbalance (Gao et al., 2019b).

#### 2.3.5 Anti-Inflammatory

**FIGURE 4:**
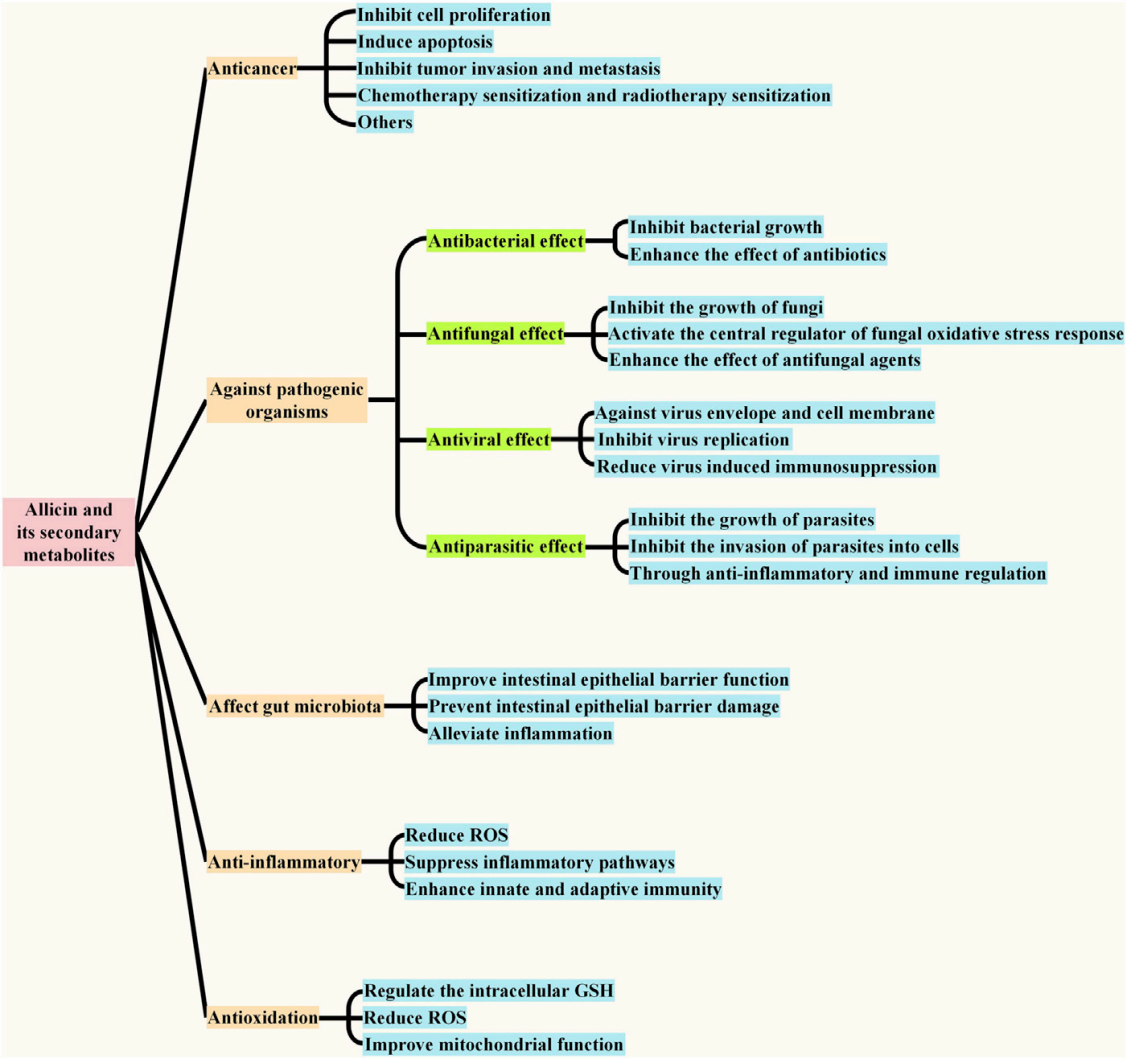
| Biological functions of allicin and its secondary metabolites. The main physiological functions of allicin and its secondary metabolites include anticancer, acting against pathogenic organisms, affecting gut microbiota, antioxidant and anti-inflammatory, and the effects against pathogenic organisms include anti-bacterial effect, anti-fungal effect, anti-viral effect, and anti-parasitic effect. The main effect of each function is summarized in the figure.

Allicin is the compound responsible for the anti-inflammatory effects of garlic (Shin et al., 2013). It has been found to reduce inflammation caused by diabetic macroangiopathy through both the Nrf2 and NF-κB pathways in mice (Li CL. et al., 2020). In another study, allicin was shown to improve osteoarthritis by downregulating PI3K/Akt/NF-κB signaling (Qian et al., 2018). It can also significantly alleviate the inflammation caused by trinitrophenylsulfuric acid by inhibiting the expression of the P38 and JNK pathways and NF-κB (Li et al., 2015). Allicin protected against inflammation by inhibiting ROS production and regulating autophagy in a mouse model infected with *Aspergillus fumigatus*, and it has also been found to improve inflammation and oxidative stress in a rabbit model infected with *Pasteurella multocida* type B (Alam et al., 2018; Dai et al., 2020). Moreover, allicin has been proven to play a hepatoprotective role against acetaminophen (APAP)-induced liver injury by reducing oxidative stress, inhibiting inflammatory pathways, and inhibiting hepatocyte apoptosis (Samra et al., 2020). Allicin could also protect against acute murine malaria infection through enhancement of the host innate and adaptive immune responses (Feng et al., 2012). In terms of psychotherapy, allicin can reduce the apoptosis of hippocampal neurons by inhibiting neuroinflammation and the NLRP3 inflammasome, thereby alleviating the depression-like behavior induced by chronic social failure stress (Gao et al., 2019a). In addition to allicin, its secondary metabolite *Z*-ajoene has also been found to have anti-inflammatory effects. *Z*-ajoene has been found to inhibit the pro-inflammatory cytokine (including IL-1 β, IL-12 and IL-6 β), and up regulate the anti-inflammatory cytokine (IL = 10) (Hitchcock et al., 2021). Moreover, it is reported that *Z*-ajoene or its analogue dansyl-ajoene was found to decrease phosphorylation and nuclear translocation of STAT3, and to covalently modify the protein by *S*-thiolation at Cys108, Cys367, and Cys687 (Hitchcock et al., 2021). In the same study, *Z*-Ajoene was also found to inhibit the activity of cyclooxygenase 2 (COX2) in a dose-dependently and non-competitively manner, which may be attributed to the *S*-thiolation at Cys9 and Cys299 (Hitchcock et al., 2021). It is reported that the anti-inflammatory effect of allicin is related to its anti-cancer effect (Schäfer and Kaschula, 2014). OSCs in garlic has been shown to inhibit the tumor-mediated pro-inflammatory activity by modulating the cytokine pattern in a way that leads to an overall inhibition of NF-κB (Schäfer and Kaschula, 2014). NF-κB is the central regulator of pro-inflammatory gene expression, and acting as the molecular link between inflammation and tumor promotion and progression (Ide and Lau, 2001). Therefore, OSCs in garlic are considered to inhibit tumor *via* acting as immune modulators that can shift the balance from a pro-inflammatory and immunosuppressive environment to an enhanced anti-tumor response (Schäfer and Kaschula, 2014) (Figure 4).

## 3 Effect of Allicin on Cancers of the Digestive System

### 3.1 Epidemiological Studies

Many epidemiological studies have been conducted to explore the effect of allium vegetables intake on digestive system cancers in the population. Though many studies have shown that the consumption of allium vegetables can reduce the risk of digestive system cancers, some studies have shown conflicting results (Kim et al., 2018; Zhou et al., 2020). We have discussed conclusions of these studies and the possible causes of the conflicting results, and summarized them as follows.

#### 3.1.1 Gastric Cancer

A recent Italian case-control study that contained 230 cases and 547 controls suggests that high allium vegetable intake is associated with reduced gastric cancer risk (the group treated with high garlic intake: OR = 0.69, 95% CI: 0.41–1.15; the group treated with more than 2 portions of onion per week: OR = 0.59, 95% CI: 0.25–1.41; the group treated with frequent use of both onion and garlic: OR = 0.70, 95% CI: 0.39–1.28) (Turati et al., 2015). In addition, the meta-analysis conducted by the same research team contains 22 case-control and four cohort studies, and the results suggested that high allium vegetable intake is associated with reduced gastric cancer risk, and the pooled RR for the highest versus lowest garlic intake was 0.60 (95% CI: 0.47–0.76) (based on 12 case-control studies) (Turati et al., 2015).

A blinded randomized placebo-controlled trial conducted in Linqu County, Shandong Province, China, compared the anti-gastric cancer effects of three interventions, including anti-HP treatment with amoxicillin and omeprazole for 2 weeks and garlic (extract and oil) and vitamin (C, E, and selenium) supplementation for 7.3 years (1995–2003) (including 3,365 residents of a high-risk region for gastric cancer in total) (Li WQ. et al., 2019). The results indicated that anti-HP treatment for 2 weeks and garlic or vitamin supplementation for 7 years can significantly reduce the risk of death due to gastric cancer for more than 22 years, and garlic supplementation showed favorable persistent effects on gastric cancer incidence and mortality during the extended follow-up of 14.7 years, which became apparent after approximately 12 years of supplementation (Li WQ. et al., 2019). Moreover, no interaction was found between HP therapy and supplementation, and combination therapy may further reduce the incidence rate and mortality of gastric cancer (Li WQ. et al., 2019).

Although many previous studies suggest that garlic consumption can reduce the risk of gastric cancer, evidence from two large prospective US cohort studies is inconsistent (Kim et al., 2018). This study suggests that there is no evidence that a high intake of garlic can reduce gastric cancer risk (Kim et al., 2018). However, because the actual active principle (allicin and its secondary products) in garlic, varies with the preparation and processing methods of garlic, the anticancer potency of different garlic preparations (including raw garlic, cooked garlic, aged garlic, garlic powder, garlic oil, garlic extract) is different (Ramirez et al., 2021). Therefore, it is understandable that epidemiological studies have reached contradictory conclusions.

#### 3.1.2 Colorectal Cancer

According to the World Cancer Research Fund/American Institute for Cancer Research (WCRF/AICR) evidence for diet, nutrition, physical activity, and colon cancer risk, garlic intake can probably decrease the risks of colon cancer, although this effect is classified as “limited-no conclusion” based on the updated evidence (Clinton et al., 2020). A recent meta-analysis of garlic intake and colorectal cancer risk consisting of 8 case–control and four cohort studies suggested that an increase in garlic intake led to a decrease in CRC risk (RR = 0.80, 95% CI: 0.69–0.91) (Zhou et al., 2020). A hospital-based matched case-control study conducted in northeastern China showed that consuming allium vegetables (such as garlic, garlic stalks, leek, onion, etc.) was associated with a reduced risk of CRC both in men and women (aORs = 0.21 when comparing high total allium intake and low total allium intake, 95% CI: 0.14–0.30, *p* < 0.001); however, this association was not significant among patients suffering from distal colon cancer (aOR = 0.53, 95% CI: 0.27–1.05, *p* = 0.248) (Wu et al., 2019).

However, a meta-analysis including eight studies of allium vegetable effects and five studies of garlic supplementation effects showed that increased intake of allium vegetables did not decrease the risk for CRC (RR = 1.06, 95% CI: 0.96–1.17, *p* = 0.26), and in the subgroup analysis of the study, the researchers found that increased intake of allium vegetables was marginally associated with increased colon cancer risk in women (RR = 1.23, 95% CI: 1.01–1.50, *p* = 0.05). In addition, they found that using garlic supplementation can increase the risk of CRC (RR = 1.18, 95% CI: 1.02–1.36, *p* = 0.03), but external validation is needed (Zhu et al., 2014). Moreover, a “use-no use” meta-analysis based on two studies showed that there was no statistically significant association between a higher garlic supplementation intake and colorectal cancer risk increase (RR = 1.24, 95% CI: 0.99–1.54) (Dorant et al., 1996; Satia et al., 2009; Heine-Bröring et al., 2015). A pooled analysis of seven cohort studies and seven case-control studies concluded that garlic consumption is not related to a reduced CRC risk (OR = 0.93, 95% CI: 0.82–1.06, *p* = 0.281; I^2^ = 83.6%, *p* ≤ 0.001) (Chiavarini et al., 2016). Another meta-analysis of five prospective cohort studies also concluded that there was no significant association between the consumption of either raw or cooked garlic (RR = 1.06, 95% CI: 0.95–1.19) or garlic supplementation (RR = 1.12, 95% CI: 0.96–1.31) and CRC risk (Hu et al., 2014).

#### 3.1.3 Esophageal Cancer

Epidemiological research on the association of garlic consumption with esophageal cancer is very limited (Kim and Kwon, 2009). A study including 395 cases (median age: 60 years) and 1,066 controls (median age: 60 years) suggested that the increase in garlic consumption was inversely correlated with the risk of esophageal cancer (OR = 0.43) (Galeone et al., 2006). An epidemiological study conducted in a Chinese smoking and drinking esophageal cancer population suggests that high raw garlic intake can reduce the risk of esophageal cancer and may act as a potential prevention factor among high-risk smokers and drinkers for esophageal cancer in the Chinese population (Jin et al., 2019). In addition, a meta-analysis of observational studies suggests that there is a moderate inverse association between allium vegetable intake and the risk of squamous cell carcinoma of the upper aerodigestive tract in case-control studies (total allium: 0.79 (95% CI 0.56–1.11), garlic: 0.74 (95% CI 0.57–0.95), and onion: 0.72 (95% CI 0.57–0.91) for the highest versus the lowest consumption) (Guercio et al., 2016).

#### 3.1.4 Other Digestive System Cancers

To explore the preventive factors for liver cancer, a large population-based case-control study conducted in Jiangsu, China, interviewed 2011 incident liver cancer cases and 7,933 randomly selected population controls from 2003 to 2010 and concluded that after controlling for known risk factors and potential confounders, such as virus infection and drinking, compared to patients who ingested no raw garlic or did so less than twice per week, those who ate raw garlic twice or more per week had reduced rates of liver cancer [adjusted odds ratio (aOR) = 0.77, 95% CI 0.62–0.96] (Liu et al., 2019). In their stratified analyses, the researchers also found that the high intake of raw garlic was inversely related to liver cancer among people without a family history of liver cancer, hepatitis B surface antigen (HBsAg)-negative individuals, frequent alcohol drinkers, and patients who had a history of eating mold-contaminated food or drinking raw water; moreover, they also identified potential additive interactions between low raw garlic intake and HBV infection or heavy alcohol drinking (Liu et al., 2019). The authors suggested that raw garlic might be used as a dietary intervention for reducing liver cancer in the Chinese population (Liu et al., 2019).

Another epidemiological study showed that allium plant intake is also associated with pancreatic cancer risk. The study included 532 cases and 1,701 age- and sex-matched controls from 1995 to 1999 and showed that the risk of pancreatic cancer was inversely associated with the consumption of total and specific vegetables and fruits, and for onions and garlic, the OR and 95% CI for the highest versus the lowest quartile were 0.46 and 0.33–0.63, respectively (Chan et al., 2005).

A population-based case-control study in Shanghai, China, assessed the relationship between diet and biliary tract cancer (Nelson et al., 2017). The researchers collected food frequency questionnaire data from 225 gallbladder cancer cases, 190 extrahepatic bile duct cancer cases, and 68 ampullae of Vater cancer cases and created 39 food groups. The allium food group, which consisted of onions, garlic, and shallots, showed an inverse association with gallbladder cancer (OR = 0.81, 95% CI 0.68–0.97) (Nelson et al., 2017). In addition, the protective effects of allium plants such as onion and garlic toward gallbladder cancer have also been observed in a case-control study including 1,170 histologically confirmed cases and 2,525 group-matched visitor controls in India (Mhatre et al., 2020).

#### 3.1.5 Conclusion for Epidemiological Studies

To sum up, the current epidemiological study on the association between allium vegetables and digestive system tumors is mainly concentrated on gastric cancer and colorectal cancer. Relatively few studies have been done on other digestive cancers, such as esophageal, liver, cholangiocarcinoma and pancreatic cancer. The evidences of epidemiological studies are not certain that the increase of allium vegetable consumption is inversely associated with the risk of gastric cancer and colorectal cancer. This is because different allium preparations have different active principle which leads to various effects. Therefore, some studies have reached contradictory results. Further study on the association between consumption of different allium vegetable preparations and the risk of gastric cancer and colorectal cancer is needed. Taking garlic as an example, studies on the association between the consumption of different garlic preparations (including raw garlic, cooked garlic, aged garlic, and garlic supplements) and the risk of gastric and colon cancer should be further conducted. The epidemiological evidence of esophageal cancer, liver cancer, cholangiocarcinoma, and pancreatic cancer has drawn a relatively consistent conclusion that the increase in consumption of allium vegetables is inversely associated with cancer risk. This may be because the epidemiological studies on the association between allium vegetables and these cancers are relatively few, and more epidemiological evidence is needed to prove the reliability of the conclusion.

### *3.2 In vitro* Studies

#### 3.2.1 Gastric Cancer

##### 3.2.1.1 Inhibition of Tumor Cell Proliferation

A study clarified the effect of allicin on the cell cycle of human gastric cancer cells and its possible mechanism by treating gastric cancer cell lines MGC-803 and SGC-7901 (two human gastric cancer cell lines) with allicin (Ha and Yuan, 2004). The results showed that allicin induced gastric cancer cell stagnation at the M stage, which may be related to the upregulation of *p21WAF1* and *p16INK4* genes (Ha and Yuan, 2004). Another study showed that allicin has an obvious inhibitory effect on the proliferation of gastric cancer cells by arresting the G2/M phase of the cell cycle of the SGC-7901 cell line (Tao et al., 2014).

A study focused on the effect of DATS toward the SGC-7901 cell line found that DATS can inhibit the growth of SGC-7901 cells and induce cell cycle arrest (Jiang X. et al., 2017). This study showed a significant decrease in the G1 phase (*p* < 0.05) and a corresponding increase in the G2/M phase (*p* < 0.05) of SGC-7901 cells treated with 200 μM DATS for 24 h compared with the control group (Jiang X. et al., 2017). Another study proved the antitumor effect of DATS in BGC-823 cells (a human gastric cancer cell line). The result showed that DATS stabilized the cell cycle at G2 phase through regulation of intracellular cyclin levels (significant accumulation of cyclin A2 and B1) to suppress cell viability (Jiang XY. et al., 2017).

##### 3.2.1.2 Induction of Apoptosis

Allicin has been found to inhibit the activity of telomerase and induce apoptosis of gastric cancer SGC-7901 cancer cells and has also been shown to induce apoptosis by arresting the G2/M phase (Sun and Wang, 2003). A study exploring the potential mechanism of allicin in gastric cancer cells showed that allicin induced SGC-7901 cancer cells apoptosis by simultaneously activating intrinsic mitochondria and extrinsic Fas/FasL-mediated apoptosis (Zhang et al., 2010). The specific effects on the two pathways were to induce the release of cytochrome C from mitochondria and increase the activation of caspase-3, -8, and -9 at the molecular level and upregulate the expression of Bax and Fas in tumor cells (Zhang et al., 2010). Moreover, in MGC-803 human gastric carcinoma cells, allicin was found to induce apoptosis of tumor cells by increasing the expression of P38 and cleaved caspase-3 through the P38 MAPK/caspase-3 signaling pathway (Zhang et al., 2015). Although these studies showed the ability of allicin to induce apoptosis of gastric cancer cells and its corresponding signal transduction mechanism, they did not clarify the specific molecular mechanism of allicin induced apoptosis of gastric cancer cells. However, studies focus on other cancers has illustrated the molecular mechanism of allicin against cancer, which may provide direction for further study of the molecular mechanism of allicin against gastric cancer (Miron et al., 2008; Li et al., 2018). Under physiological conditions, allicin can easily penetrate the cell membrane and react with sulfhydryl groups (e.g., GSH cysteine residues) to produce oxidized biomolecules. In leukemia cells (HL-60 and U937), the reaction of allicin and GSH is the trigger of apoptosis (Li et al., 2018). Allicin (5 µM) rapidly moved inside the cells and oxidized GSH in glutathione disulfide (GSSG), which lead to the GSH/GSSG ratio imbalance and cellular reduction potential subsequently decrease, then causing mitochondrial damage and starting the intrinsic apoptotic pathway (Miron et al., 2008).

Allicin exerts its anti-cancer activity *via* the thiol-disulfide exchange reaction (TDER) with protein thiols (Poole, 2015). DATS can also undergo TDER with protein thiols, and additionally produce H_2_S gas *via* reacting with the cellular thiol glutathione, which can regulate various processes in the cells (Rose et al., 2018). DATS can induce apoptosis of cancer cells by regulating apoptosis-related proteins as well as activating MAPK and affecting PI3K/AKT pathways in cells (Jiang X. et al., 2017). The results showed that DATS not only downregulated the anti-apoptotic factor Bcl-2 but also increased the expression of P53 and cytochrome C (apoptosis markers) (Jiang X. et al., 2017). Furthermore, DATS has been found to activate three MAPK pathways, including the ERK, JNK, and P38 pathways, in SGC-7901 cells (Jiang X. et al., 2017). In another gastric cancer cell line, BGC-823, the growth inhibitory effect of DATS has also been suggested to correlate with apoptosis due to the obvious apoptosis sub-G_1_ peak in cell cycle analysis (Jiang XY. et al., 2017). In this study, DATS significantly downregulated Bcl-2 and upregulated Bax and the levels of P53 and cytochrome C (Jiang XY. et al., 2017). The activation of cysteine proteases is an important pathway in cell apoptosis, and this study demonstrated that DATS can induce apoptosis in BGC-823 cells through activation of the caspase pathway, attenuation of Nrf2/Akt, and activation of the JNK and P38-MAPK pathways (Olsson and Zhivotovsky, 2011; Jiang XY. et al., 2017).

##### 3.2.1.3 Anti-Helicobacter pylori

At present, the research on the anti-HP effect of allicin is still limited. A study focused on the effect of garlic oil on HP in the environment of artificial gastric juice both alone and in the presence of other substances such as mucus, peptone, rapeseed oil, dextrin, and simulated meal mixtures, and the researchers concluded that although the anti-HP activity of garlic oil was affected by food materials and mucin, it still retained high activity under simulated gastric conditions (O'Gara et al., 2008).

#### 3.2.2 Colorectal Cancer

##### 3.2.2.1 Inhibition of Tumor Cell Proliferation

The antiproliferation effect of allicin may be attributed to the ability of allicin to instantaneously deplete intracellular GSH levels (Hirsch et al., 2000). It is reported that allicin can easily penetrate the cell membrane and react with the cellular thiol glutathione *via* thiolysis exchange producing *S*-allylmercaptogluthione, to transiently deplete the intracellular GSH level, and induce the inhibition of cell cycle progression and growth arrest (Hirsch et al., 2000). The same study also reported that the extent of reduction in GSH level was related to allicin-induced growth inhibition (Hirsch et al., 2000). Allicin has been reported to induce ROS in cultured cancer cells in a dose-dependent manner (Schäfer and Kaschula, 2014). However, GSH in cancer cells quenches ROS produced by allicin, and the rapid reduction in GSH may allow the excess of allicin to react directly with different thiol-containing molecules in the cell that are usually protected by GSH (Hirsch et al., 2000). Allicin has been found to reduced human colon cancer cell activity and inhibited cell proliferation in a concentration-dependent manner on HT-29 cell line (Gruhlke et al., 2016). It has been shown to have a time- and dose-dependent cytostatic effect on the proliferation of HCT-116, LS174T, and Caco-2 colon cancer cell lines, at concentrations ranging from 6.2 to 310 µM (Bat-Chen et al., 2010). It has also been observed to block the cell cycle to exert it s anti-proliferation effect on LoVo human colon cancer cells (Gao et al., 2009a).

Accumulating evidence has shown that ABGE might prove beneficial in preventing or inhibiting oncogenesis through inhibiting the growth of cancer cells and inducing apoptosis, and the mechanism is to inhibit the PI3K/Akt pathway (Dong et al., 2014). ABGE upregulates PTEN, downregulates Akt and p-Akt expression, and suppresses the downstream target of the PI3K/Akt pathway (70-kDa ribosomal protein S6 kinase 1) at mRNA and protein levels (Dong et al., 2014). AGE has also been shown not only to significantly inhibit the proliferation of human colorectal carcinoma cell lines, including HT29, SW480, and SW620 cells, but also to suppress the growth of endothelial cells, including ECV304 cells and TRLECs (transformed rat lung endothelial cells), which indicates that AGE can inhibit angiogenesis (Matsuura et al., 2006). Compared with endothelial cells, colorectal carcinoma cells seemed to be suppressed at slightly lower concentrations of AGE (Matsuura et al., 2006). Finally, the researchers concluded that AGE can directly suppress the proliferation of colorectal carcinoma cells and inhibit tumor angiogenesis (Matsuura et al., 2006). Furthermore, the molecular mechanism of the antitumor proliferation effect of AGE was explored in another study. The results showed that AGE delayed cell cycle progression by downregulating cyclin B1 and CDK1 expression of NF-κB in human colorectal cancer cells but did not induce apoptosis (Jikihara et al., 2015). Another study investigated the effects of CGE on the proliferation of the human cancer cell lines and mouse macrophage cell line (TIB-71), including hepatic (Hep-G2), colon (Caco-2), prostate (PC-3), and breast (MCF-7) cell lines (Bagul et al., 2015). The authors found that for Hep-G2, MCF-7, TIB-71, and PC-3 cells, the inhibition of cell proliferation reached 80–90% (treated with 0.125, 0.25, 0.5, or 1 μg/ml of CGE), while for the Caco-2 cells, the inhibition was only 40–55% (treated with 0.25, 0.5, or 1 μg/ml) (Bagul et al., 2015). However, in the coculture study of Caco-2 and TIB-71 cells, the proliferation inhibition rate for Caco-2 cells was 90%, compared to 40–55% when cultured separately (Bagul et al., 2015).

##### 3.2.2.2 Induction of Apoptosis

Allicin has been shown not only to block the cell cycle but also to induce cell apoptosis in LoVo human colon cancer cells (Gao et al., 2009a). Moreover, a study investigated the effects of OSCs in garlic, including allicin, and its secondary metabolites, on human Caco-2 and HT-29 colon carcinoma cell lines and showed that OSC-induced cell death in the two cell lines in the following order: allicin < DAS = DADS < DATS (Caco-2 cell line) and allicin = DAS < DADS < DATS (HT-29 cell line) (Jakubíková and Sedlák, 2006). It was concluded that the number of sulfur atoms in OSCs was correlated with their ability to induce apoptosis, and the result also supported the role of redox-sensitive “sulfhydryl switches”, which are commonly triggered by disulfide bond formation and can also be controlled by *S*-thiolation, in maintaining the intracellular redox milieu (Jakubíková and Sedlák, 2006). Allicin reacts with protein thiols *via* undergoing TDER and modifies the cell protein to exert its anti-cancer activity (Poole, 2015). A study proved the cytotoxic effects of allicin purified from fresh garlic cloves on HCT116 colon cancer cells and showed that allicin induced apoptosis *via* a mechanism associated with transactivation of the transcription factor Nrf2 and was characterized by decreasing Bcl-2 levels, increasing Bax levels, enhancing hypodiploid DNA content, and enhancing the ability to release cytochrome C from mitochondria to the cytosol (Bat-Chen et al., 2010). In addition to allicin, as mentioned above, DATS also reacts with protein thiols *via* TDER and additionally produce H_2_S. It acts as a fast H_2_S donor in the cell, and H_2_S has been reported that can induce apoptosis of cancer cells (Liang et al., 2015; Lee et al., 2017). As a gas, H_2_S can react quickly, which also explained why DATS is the most active OSC in inducing apoptosis in previous studies. Moreover, in HT-29 colon cancer cells, DATS has been found to oxidative modify thiols of specific cysteine residues (Cys12 β and Cys354 β) in β-tubulin molecules, thereby inducing rapid microtubule disassembly, and inducing apoptosis of HT-29 colon cancer cells (Hosono et al., 2008).

##### 3.2.2.3 Inhibition of Tumor Invasion and Metastasis

Allicin has been shown to inhibit the invasion and metastasis of LoVo human colon cancer cells by downregulating the expression of VEGF, u-PAR, and HPA mRNA (Gao et al., 2009b). VEGF is an important tumor angiogenesis factor related to tumor vascularization, metastasis, and growth; in addition, both u-PAR and HPA can also promote tumor invasion and metastasis (Andreasen et al., 2000; Boyd and Nakajima, 2004; Gao et al., 2009b). Therefore, allicin decreases the expression of VEGF, u-PAR, and HPA, indicating that it can inhibit the invasion and metastasis of tumors. AGE can inhibit cell motility and invasion by enhancing the adhesion of endothelial cells to collagen and fibronectin and inhibit angiogenesis *via* the suppression of endothelial cell motility and proliferation, as well as the tube (which can later become blood vessels) formation of endothelial cell (Matsuura et al., 2006). The study found that AGEs could inhibit the invasiveness of SW480 and SW620 cells but had no effect on the invasive activity of HT29 cells, suggesting that AGEs’ anti-invasiveness appears to be dependent on the type of cancer cell (Matsuura et al., 2006).

##### 3.2.2.4 Chemosensitization

It has been reported that allicin can enhance the cytotoxicity of CPT-11 (an inhibitor of Topoisomerase I, which is used as first-line chemotherapy for advanced or metastatic colorectal cancers) (Gao et al., 2009a; Si J. et al., 2019). Allicin has also been found to increase the impact of 5-FU and oxaliplatin (500 µM) in decreasing the *via*bility of colon cancer cells, has a better effect than standard 5-FU and oxaliplatin chemotherapy, and reduces the clinical cost of treatment at the same time (Perez-Ortiz et al., 2020).

#### 3.2.3 Liver Cancer

##### 3.2.3.1 Induction of Autophagy in Cancer Cells

Allicin can induce P53-mediated cell autophagy and inhibit the *via*bility of the human hepatocellular carcinoma cell line HepG2 (Chu et al., 2012). The P53 protein is a tumor suppressor protein, and it has been found to participate in cellular autophagy in various pathways and ultimately result in the modulation of mTOR, AMPK, and TSC2 (Chu et al., 2012). Autophagy regulation of P53 has a dual effect, promoting autophagy in the nucleus while suppressing autophagy in the cytoplasm (Sui et al., 2011). Allicin has been found to decrease the levels of cytoplasmic P53, Bcl-2, and the PI3K/mTOR signaling pathway and to increase the expression of the AMPK/TSC2 and Beclin-1 signaling pathways in HepG2 cells, which can also induce the degradation of mitochondria in cancer cells, therefore, allicin promote the autophagy of cancer cells (Chu et al., 2012). It has also been reported that allicin could regulate autophagic cell death pathway through *p53* gene in both transcription and translation levels, and the existence of *p53* may guide cancer cells to autophagy or lead other cancer cells without *p53* to apoptosis (Chu et al., 2013).

##### 3.2.3.2 Protection Effect on Hepatocytes and Chemosensitization Effect on Cancer Cells

Allicin and secondary metabolites can protect cells from damage caused by some carcinogens or chemotherapy. The antigenotoxic activity of several OSCs in garlic (including allicin, DAS, DADS, SAC, and AM) has been assessed in the human hepatocellular carcinoma cell line HepG2 by using the comet assay, and it has been found that all the studied OSCs were shown to reduce the genotoxicity of the direct-acting compounds hydrogen peroxide and methyl methanesulfonate (Belloir et al., 2006). In addition, except for AM, all the studied OSCs were found to significantly decrease the genotoxicity of aflatoxin B1 (Belloir et al., 2006). Another study showed that allicin can also enhance the cytotoxicity induced by 5-fluorouracil (5-FU) (anticancer drug) in HCC cells by the ROS-mediated mitochondrial pathway, and this may provide a novel chemotherapy regimen for HCC (Zou et al., 2016).

#### 3.2.4 Other Digestive System Cancers

Allicin has been shown to suppress the proliferation of cholangiocarcinoma cells by activating the caspase cascade, inducing apoptosis, and reducing the expression of STAT3 downstream proteins such as Bcl-2 (Chen et al., 2018). In addition to cholangiocarcinoma, OSCs have also been found to induce apoptosis in pancreatic cancer cells. Garlic oil has been observed to inhibit the proliferation of pancreatic cancer cell lines, including AsPC-1, PANC-1, and Mia PaCa-2 cells, and a study suggested that garlic oil could induce a pro-apoptosis effect on AsPC-1 cells (Lan et al., 2013). One of the main components of garlic oil, DATS, has been shown to induce apoptosis in pancreatic cancer cells (Capan-2) as well as nontumorigenic pancreatic ductal epithelial cells (H6C7), and it has been observed that DATS could increase the G2/M phase in the cell cycle through increasing cyclin B1 and p21 levels and decreasing cyclin D1 level (Ma et al., 2014).

#### 3.2.5 Stability

To better understand the anticancer effect of allicin and DATS, we should also pay attention to their stability. Under the influence of temperature, allicin can easily degrade to form a variety of compounds, including DATS, ajoenes and vinyldithiins (El-Saber Batiha et al., 2020). Allicin has been observed to be more stable in protic polar methanol than in aprotic polar ethyl acetate. It has also been reported that about 90% of the allicin remained in the stimulated intestinal fluid (pH = 7.5) and simulated gastric fluid (pH = 1.2) after incubation at 37 °C for 5 h, while only a small amount of allicin could be detected after incubation in blood for 5 min (Freeman and Koder, 1995). It is also reported that the biological and chemical half-lives of allicin in aqueous and ethanolic solutions at room temperature are about 6 and 11 days, respectively, while in vegetable oil, allicin is unstable with an activity half-life of 0.8 h and a chemical half-life of 3.1 h (Fujisawa et al., 2008). Moreover, it has been shown that acidic media were more conducive than neutral or alkaline media to stabilize thiosulfinates (e.g. allicin), and allicin formed at an optimum pH of 4.5–5.0 (Lawson and Hughes, 1992; Shen et al., 2002). DATS is the secondary metabolite of allicin, and has also been found to degrades rapidly under normal conditions, therefore, some suitable DATS delivery systems have been conducted to better evaluate its anti-cancer effect (Ju et al., 2010; Janik-Hazuka et al., 2021).

#### 3.2.6 Conclusion of *in Vitro* Studies

To sum up, both allicin and DATS have showed anti-digestive system cancer activity. They exert anti-tumor activity mainly by inhibiting proliferation, inducing apoptosis, suppressing migration and metastasis, and enhancing chemosensitivity of the tumor cells. *In vitro* evidences have shown that allicin is mainly against gastric cancer, colorectal cancer, liver cancer and cholangiocarcinoma, while DATS is mainly against gastric cancer, colorectal cancer and pancreatic cancer. To date, there is no evidence of allicin and DATS against esophageal cancer *in vitro*. It is reported that ajoene, another secondary product of allicin, and its analogues have anti-esophageal cancer effects (Kaschula et al., 2011). The basis of allicin and DATS exerting their anti-cancer ability is the thiol-disulfide exchange reaction. Allicin quickly moves inside the cells and reacts with protein thiol groups to modify cell protein and affect signaling, while DATS not only reacts with protein thiols but also intracellularly produces H_2_S *via* TDER, and then exerts subsequent effects on a series of processes such as modifying cell protein, signaling, and metabolism through H_2_S.

**FIGURE 5:**
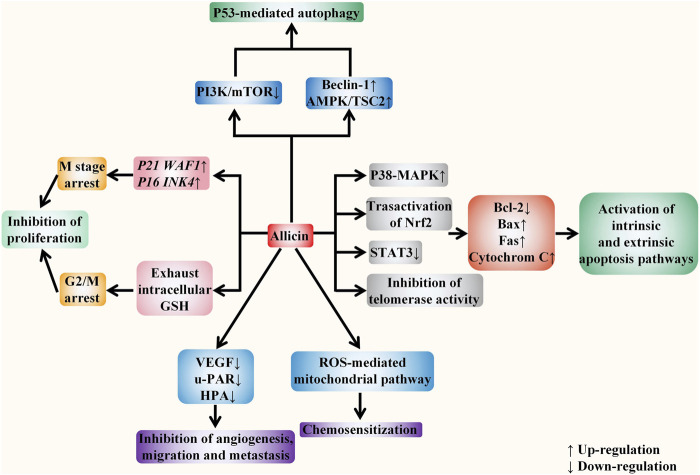
Allicin acts against digestive system cancers *in vitro*. The anticancer effect of allicin *in vitro* is mainly reflected in five aspects, including inhibiting proliferation, inducing apoptosis, inducing autophagy, inhibiting angiogenesis, invasion, and metastasis, and enhancing the sensitivity of tumor chemotherapy. It depletes intracellular glutathione (GSH) and up-regulates *p21WAF1* and *p16INK4* genes to block the cell cycle and inhibit cell proliferation. It activates both endogenous and exogenous apoptotic pathways by regulating intracellular signaling pathways, inhibiting telomerase activity, and regulating the activity of apoptosis-related proteins. In addition, it also down-regulates the PI3K/mTOR signaling pathway and up-regulates the AMPK/TSC2 and Beclin-1 signaling pathways to induce the P53 mediated autophagy. It inhibits tumor angiogenesis, migration, and metastasis by down-regulating VEGF, u-PAR, and HPA. Moreover, *via* ROS -mediated mitochondrial pathway, it exerts the chemosensitization effect towards tumor cells.

**FIGURE 6:**
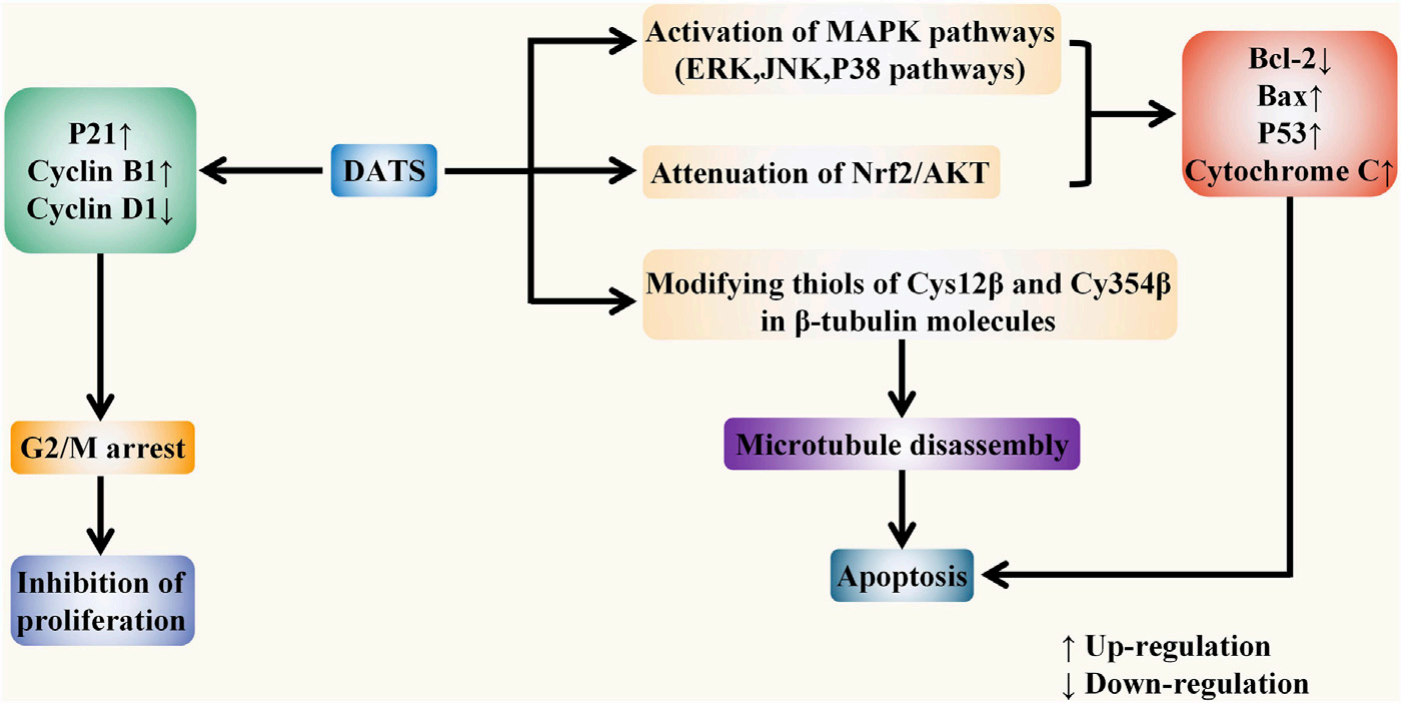
DATS fights against digestive system cancers *in vitro*. DATS exerts the anti-tumor role mainly in two ways: inhibiting proliferation and inducing apoptosis. DATS can inhibit cell proliferation by inducing G2/M phase arrest *via* regulating the levels of P21 and Cyclin. In addition, DATS can activate three MAPK pathways and attenuate Nrf2/Akt pathway to regulate apoptosis-related proteins. Moreover, it has also been shown to induce rapid microtubule disassembly by modifying thiols of Cys12 β and Cys354 β in β-tubulin molecules, thereby inducing apoptosis.

The specific targets of allicin on digestive system cancer are summarized as follows. Firstly, allicin can inhibit the proliferation of cancer cells by exhausting GSH in cells. It can also block the cell cycle and inhibit cell proliferation by up-regulating *p21WAF1* and *p16INK4* genes. Secondly, allicin has been found to induce both extrinsic and intrinsic apoptosis by up-regulating the P38-MAPK pathway, transactivating Nrf2, and down-regulating the STAT3 pathway. It can also inhibit telomerase activity to induce apoptosis. Thirdly, allicin has also been shown to induce P53-mediated autophagy by down-regulating the PI3K/mTOR signaling pathway and up-regulating the AMPK/TSC2 and Beclin-1 signaling pathways. Moreover, allicin can inhibit tumor angiogenesis, migration, and metastasis by down-regulating VEGF, u-PAR, and HPA. Finally, it has a chemosensitization effect when treated with chemotherapeutic drugs including CPT-11, 5-FU, and oxaliplatin. (Figure 5). *In vitro* evidences showed that the specific anti-cancer effect of DATS is mainly reflected in inhibiting proliferation and inducing apoptosis. It arrests the cell cycle to G2/M phase by regulating cyclin and p21 levels, so as to inhibit cell proliferation. DATS has been found to induce apoptosis *via* activating three MAPK pathways (including the ERK, JNK, and P38 pathways), and attenuating Nrf2/Akt pathway, as well as regulating apoptosis-related proteins. Moreover, it has also been shown to inducing apoptosis via rapid microtubule disassembly by modifying thiols of Cys12 β and Cys354 β in β-tubulin molecules. (Figure 6). AGE has also been shown to suppress proliferation, inhibit tumor angiogenesis, and inhibit tumor invasion and metastasis. In conclusion, the anti-tumor effect of allicin, DATS, and garlic extract on digestive system *in vitro* is noteworthy.

### 3.3 *In vivo* Studies

#### 3.3.1 Gastric Cancer

In SGC-7901 xenograft mice, it has been found that DATS can significantly suppress tumor growth, which was manifested in the following aspects (Jiang X. et al., 2017). Firstly, it significantly decreased the volume (*p* < 0.001) and the weight (*p* < 0.001) of the tumor (Jiang X. et al., 2017). Secondly, it stimulated the immune response of the host organism by increasing the levels of IL-12, IFN-γ, and TNF-α, which can also be found in mice without xenograft (Jiang X. et al., 2017). Thirdly, it also blocked proliferation, as well as inducing apoptosis of tumors through activating MAPK pathways (Jiang X. et al., 2017). Moreover, it suppressed tumor invasion and metastasis by regulating the expression of MMP-9 (participating in remodeling extracellular matrix) and E-cadherin (a cell adhesion junction molecule), without significant side effects in major organs of the SGC-7901 xenograft mice (Jiang X. et al., 2017). The antitumor effect of DATS has also been proved in a BALB/c nude mouse BGC-823 xenograft model. The results suggested that DATS can slow tumor growth and reduce the weight and volume of tumors compared to the control group (*p* < 0.01 for both), and it can induce apoptosis and downregulate Ki-67 (proliferation marker) expression in tumor tissues (Jiang XY. et al., 2017). The study also showed that DATS can significantly activate kinases such as P38 and JNK/MAPK and attenuates the Nrf2/Akt pathway in the BGC-823 xenograft mouse model (Jiang XY. et al., 2017). Moreover, the study indicated that DATS can improve the antitumor efficacy of cisplatin (DDP, a chemotherapeutic agent widely used in the treatment of solid malignant tumors) in the treatment of gastric cancer without notable side effects (Jiang XY. et al., 2017).

A clinical trial focused on the effect of local application of allicin under gastroscope among patients with gastric adenocarcinoma showed that local application of allicin could inhibit cell proliferation and induce apoptosis of progressive gastric carcinoma through increasing Bax and Fas expression and decreasing the Bcl-2 expression level (Zhang et al., 2008). The clinical trial results of allicin against HP are controversial. Although the anti-H. pylori activity of garlic powder and garlic oil has been proved in an *in vitro* study, subsequent clinical studies failed to confirm this activity (McNulty et al., 2001; Si XB. et al., 2019). However, allicin has been found to improve HP eradication, healing of ulcers, and remission of symptoms and was suggested to have potential as an add-on therapy (Si XB. et al., 2019). In addition, ABGE has been proved to induce dose-dependent apoptosis in SGC-7901 human gastric cancer cells, and further study in tumor-bearing mice revealed that ABGE could significantly inhibit the growth of the tumor due to its antioxidant and immunomodulatory effects (Wang et al., 2012).

#### 3.3.2 Colorectal Cancer

DATS is also considered to be a potential anti-colorectal cancer agent. Based on the fact that DATS can inhibit the growth of tumor cells *in vitro* by blocking the cell cycle, destroying the cell microtubule network, increasing caspase-3 activity, and inducing apoptosis, the researchers conducted *in vivo* experiments, and the results showed that DATS can also suppress tumor growth in nude mice bearing HCT-15 xenografts (Hosono et al., 2005). Allicin has been proved to promote HCT116 cell apoptosis and suppress cancer cell survival and proliferation by inhibiting the STAT3 signal, and this result has also been confirmed in the azoxymethane methane/sodium dextran sulfate (AOM/DSS) model of colorectal cancer mice (Li X. et al., 2019). In addition, a further study found that allicin could improve the radiosensitivity of colorectal cancer cells by inhibiting the NF-κB signaling pathway in the BALB/c mouse model, which suggests that allicin may be used as a potential sensitizer in clinical tumor radiotherapy (Huang et al., 2020). In addition to treatment with allicin or its secondary metabolites alone, a double-blind randomized clinical trial suggested that using AGE can reduce the occurrence, growth, and spread of colorectal adenomas (Tanaka et al., 2006).

#### 3.3.3 Liver Cancer

Both allicin and DATS have been shown to exert anti-hepatocellular carcinoma effect in the mice model (Zhang et al., 2006; Zhang et al., 2007). On BALB/c mice xenografted with BEL7402 hepatocellular carcinoma cells, it was found that allicin can up-regulate the expression of Bax and FasL in a dose-dependent manner to induce the apoptosis of tumor cells (Zhang et al., 2006). Allicin has also been shown to suppress the growth of tumor volume in a dose-dependent manner, and at the concentration of 5 mg/ml, allicin showed a stronger effect on inhibiting the growth of tumor volume than that of the positive control group (treated with 0.2 mg/mLl adriamycin) (Zhang et al., 2006). Moreover, it was also observed that combination of low-dose allicin (1 mg/ml) and adriamycin (0.2 mg/ml) has better inhibitory effect on tumor than single drug treatment of high-dose allicin (5 mg/ml), suggesting the ability of allicin to be used as an adjuvant compound in chemotherapy. In addition, allicin has alos been found to promote the antitumor activity of 5-FU toward HCC xenograft tumors in nude mice by increasing intracellular ROS level, reducing mitochondrial membrane potential (ΔΨm), activating caspase-3 and PARP, and downregulating Bcl-2 (Zou et al., 2016).

On orthotopic transplantation HepG2 hepatocellular carcinoma model in nude mice, hepatic targeted polybutylcyanoacrylate nanoparticles of diallyl trisulfide (DATS-PBCA-NP) showed significant anti-tumor effect with good prolonged release effect and hepatic-targeted activity (Zhang et al., 2007). Treatment with DATS-PBCA-NP can dramatically decrease the expression of Bcl-2 protein without significantly changing the expression of Fas, FasL and Bax protein, causing a decrease in Bcl-2/Bax ratio, thus leading to the predominance of pro-apoptosis protein in the ratio between pro- and anti-apoptosis subsets, then resulting in apoptosis (Zhang et al., 2007).

OSCs in garlic have also been found to resist drug-induced liver cancer and liver injury. AGE has been shown to against hepatotoxicity, oxidative stress, and hepatocarcinoma induced by p-dimethylaminoazobenzene and phenobarbital in experimental rats (Pathak et al., 2020). Garlic powder has been found to reduce the DNA damage of rat liver caused by N-nitrosodimethylamine (NDMA) and aflatoxin B1, and the experimental results showed that the increase in alliin content in garlic powder is closely related to the proportional decrease in DNA alteration induced by NDMA but is not related to the decrease in liver DNA damage caused by aflatoxin B1 (Singh et al., 2006).

#### 3.3.4 Other Digestive System Cancers

Allicin has been shown to inhibit the proliferation, migration, invasion, and epithelial-mesenchymal transformation (EMT) of cholangiocarcinoma in an *in vivo* model (Chen et al., 2018). In C57/BL6 nude mouse pancreatic cancer xenograft models, researchers found that treatment with allicin alone or in combination with recombinant interleukin-2 could inhibit the progression of pancreatic tumors, and they found that the combination treatment could significantly inhibit the growth of xenografts and prolong the survival time through the activation of CD4 T, CD8 T, and NK cells (Wang et al., 2013). In addition to the antitumor effect, it has been proved that AGE can improve immune function in patients with gastrointestinal cancer (Ishikawa et al., 2006). The study included 42 patients with liver cancer, seven patients with pancreatic cancer, and one patient with colon cancer and found that treatment with AGE significantly increased both the number and the activity of NK cells without adverse effects (Ishikawa et al., 2006).

#### 3.3.5 Conclusion of *in Vivo* Studies

**FIGURE 7:**
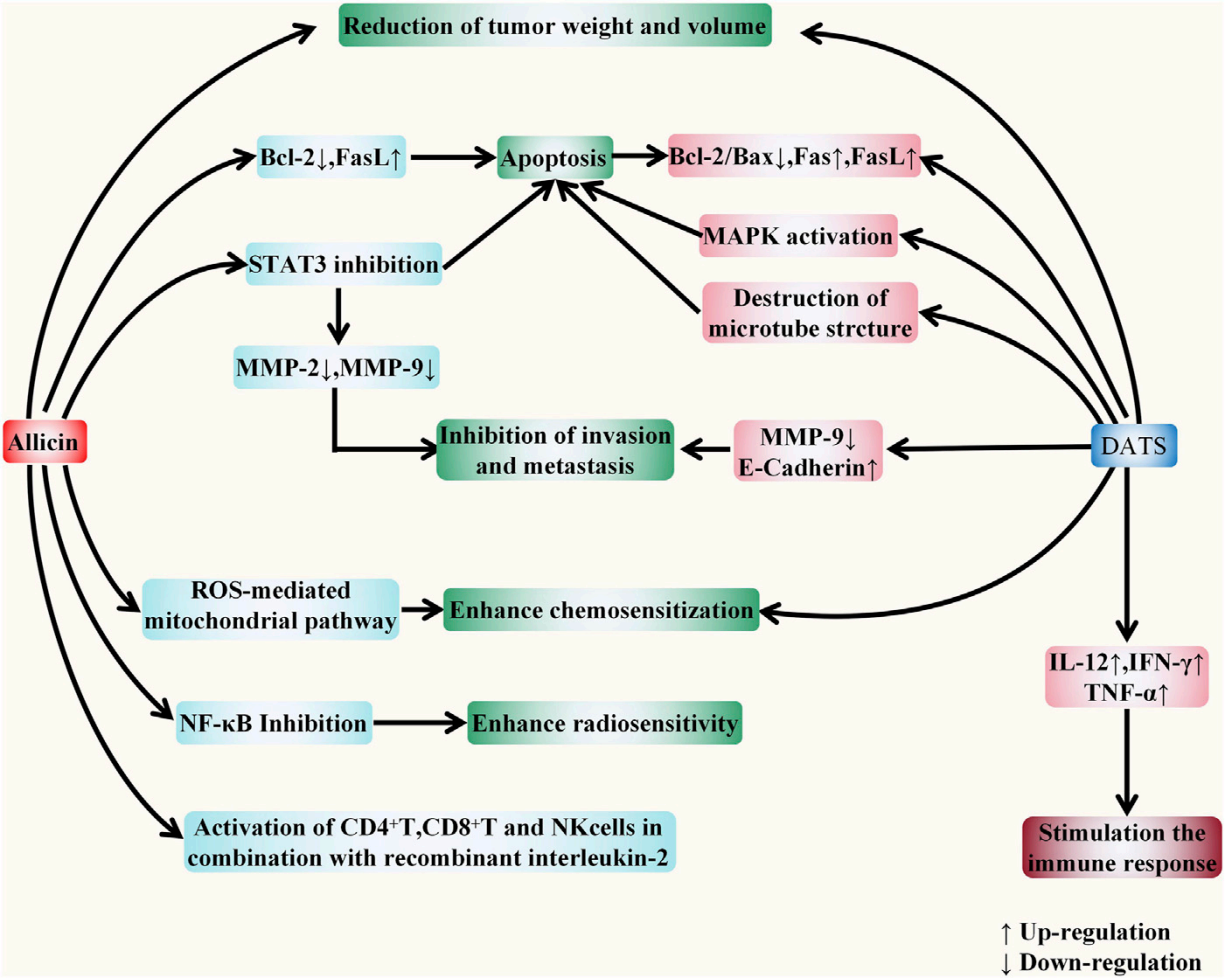
The comparison between the effects of allicin and DATS against digestive system cancers *in vivo*. The figure summarizes the different anticancer effects of allicin and DATS *in vivo*. Firstly, allicin and DATS have four consistent anticancer effects, including reducing tumor weight and volume, inducing apoptosis, inhibiting tumor invasion and metastasis, and enhancing tumor cell chemosensitivity. Both compounds can induce apoptosis by regulating apoptosis-related proteins. In addition, allicin can also induce apoptosis, inhibit tumor invasion and metastasis by inhibiting STAT3, and enhance tumor chemosensitivity through ROS-mediated mitochondrial pathway. However, DATS induces apoptosis by activating MAPK pathway and destroying cell microstructure, and inhibit invasion and metastasis by down regulating MMP-9 and up regulating E-cadherin. Allicin also has two more effects: enhancing the radiotherapy sensitivity of tumor cells and activating T cells and NK cells with the participation of recombinant interleukin-2. DATS has one more effect: increasing the expression of cytokines, including IL-12 and IFN-γ, TNF-α, to stimulate the immune response of the host.

In mice models, allicin can inhibit gastric cancer, colorectal cancer, liver cancer, cholangiocarcinoma, and pancreatic cancer, while DATS can inhibit gastric cancer, colon cancer, and liver cancer. To sum up, previous studies have shown that both allicin and DATS could play an anti-tumor role in mice models *via* reducing the weight and volume of the tumor, inducing apoptosis and proliferation, suppressing invasion and metastasis, and improving the chemosensitivity of the tumor. In addition, allicin has been found to enhance the tumor radiosensitivity, as well as activate CD4 T, CD8 T, and NK cells in combination with recombinant interleukin-2. Moreover, DATS also exert it anti-cancer ability *via* stimulate the host immune response. The effects of allicin and DATS against digestive system cancer have been shown in Figure 7. This is roughly consistent with *in vitro* evidences.

## 4 Conclusion

This review describes the synthesis process, structure, toxicity, and biological effects of allicin (also mentions the structure and biological effects of some allicin secondary metabolites e.g., DATS, *Z*-ajoene.) in section 2. Based on the chemical structure and biological effects, we summarize the role of allicin, its secondary metabolite DATS and garlic extracts (including AGE, ABGE etc.) in cancers of digestive system from the evidence of epidemiology, *in vitro* and *in vivo*. Among them, there are relatively more studies on gastric cancer, colorectal cancer and liver cancer, while relatively few studies on other digestive system cancers.

To date, allicin, DATS, and garlic extract have been shown certain anti-cancer effects on gastric cancer, colorectal cancer, liver cancer, cholangiocarcinoma, and pancreatic cancer. Their various biological activities, including anti-inflammatory, anti-oxidation, antibacterial, and anti-virus effects, also promote the anti-cancer effect. In general, the thiol-disulfide exchange reaction is the key reaction for allicin and DATS to exert their biological activities. Allicin regulates signaling and various metabolic processes in the cell by reacting with GSH and the thiols available in proteins through *S*-thioallylation. DATS reacts with cellular thiol glutathione through the thiol-disulfide exchange reaction and produces H_2_S gas to exert various regulatory functions. Base on this chemical mechanism, allicin and DATS play an anti-cancer role mainly by inhibiting tumor proliferation, inducing apoptosis, inhibiting tumor invasion and metastasis, and enhancing the chemosensitivity of tumor cells. However, this reaction is not selective, allicin can also react with protein thiols in normal cells, which indicates the cytotoxic potential and toxicity of normal cells.

Both *in vivo* and *in vitro*, allicin not only showed a direct anti-tumor effect on the digestive system but also showed a sensitization effect toward radiotherapy and chemotherapy, as well as protecting normal cells against carcinogens. However, there is no FDA-approved drug containing purified allicin. The data of allicin clinical trials are relatively lacking, and more patient-centered, multi-center and large-scale studies are needed to determine the efficacy of allicin and its secondary metabolites. Although many garlic preparations have been proved to have antitumor effects, the main components of each preparation are different, so their anticancer effects may be quite different. However, at present, there are few clinical studies on the difference of anticancer effect of allicin and its secondary metabolites, so it is difficult to determine which OSC plays the greatest anticancer effect in clinical application. In addition, the dosage and the administration method of allicin in the experiment is in lack of a unified standard. Although allicin can be used as a drug everyday by ingesting together with food, further research is needed to achieve the precise treatment of allicin against digestive system tumors. In conclusion, allicin and its secondary metabolites have shown remarkable anticancer potential, but it is still far away from its clinical application as a drug, and more experiments are needed to achieve its precise treatment of digestive system cancers.
